# Hepatocyte-specific glucose-6-phosphatase deficiency disturbs platelet aggregation and decreases blood monocytes upon fasting-induced hypoglycemia

**DOI:** 10.1016/j.molmet.2021.101265

**Published:** 2021-06-04

**Authors:** Anouk M. La Rose, Venetia Bazioti, Joanne A. Hoogerland, Arthur F. Svendsen, Anouk G. Groenen, Martijn van Faassen, Martijn G.S. Rutten, Niels J. Kloosterhuis, Bertien Dethmers-Ausema, J. Hendrik Nijland, Gilles Mithieux, Fabienne Rajas, Folkert Kuipers, Michaël V. Lukens, Oliver Soehnlein, Maaike H. Oosterveer, Marit Westerterp

**Affiliations:** 1 Department of Pediatrics, University Medical Center Groningen, University of Groningen, Groningen, the Netherlands; 2 European Research Institute for the Biology of Ageing, University Medical Center Groningen, University of Groningen, Groningen, the Netherlands; 3 Department of Laboratory Medicine, University Medical Center Groningen, University of Groningen, Groningen, the Netherlands; 4 Université Claude Bernard Lyon 1, Université de Lyon, INSERM UMR-S1213, Lyon, France; 5 Institute for Experimental Pathology (ExPat), Center for Molecular Biology of Inflammation (ZBME), University of Münster, Münster, Germany; 6 Department of Physiology and Pharmacology (FyFa), Karolinska Institutet, Stockholm, Sweden

**Keywords:** Glycogen storage disease type 1a, Hypoglycemia, Corticosterone, Monocytes, Platelets

## Abstract

**Objective:**

Glycogen storage disease type 1a (GSD Ia) is a rare inherited metabolic disorder caused by mutations in the glucose-6-phosphatase (*G6PC1*) gene. When untreated, GSD Ia leads to severe fasting-induced hypoglycemia. Although current intensive dietary management aims to prevent hypoglycemia, patients still experience hypoglycemic events. Poor glycemic control in GSD Ia is associated with hypertriglyceridemia, hepatocellular adenoma and carcinoma, and also with an increased bleeding tendency of unknown origin.

**Methods:**

To evaluate the effect of glycemic control on leukocyte levels and coagulation in GSD Ia, we employed hepatocyte-specific *G6pc1* deficient (L*-G6pc*^*−/−*^) mice under fed or fasted conditions, to match good or poor glycemic control in GSD Ia, respectively.

**Results:**

We found that fasting-induced hypoglycemia in L*-G6pc*^*−/−*^ mice decreased blood leukocytes, specifically proinflammatory Ly6C^hi^ monocytes, compared to controls. Refeeding reversed this decrease. The decrease in Ly6C^hi^ monocytes was accompanied by an increase in plasma corticosterone levels and was prevented by the glucocorticoid receptor antagonist mifepristone. Further, fasting-induced hypoglycemia in L*-G6pc*^*−/−*^ mice prolonged bleeding time in the tail vein bleeding assay, with reversal by refeeding. This could not be explained by changes in coagulation factors V, VII, or VIII, or von Willebrand factor. While the prothrombin and activated partial thromboplastin time as well as total platelet counts were not affected by fasting-induced hypoglycemia in L*-G6pc*^*−/−*^ mice, ADP-induced platelet aggregation was disturbed.

**Conclusions:**

These studies reveal a relationship between fasting-induced hypoglycemia, decreased blood monocytes, and disturbed platelet aggregation in L*-G6pc*^*−/−*^ mice. While disturbed platelet aggregation likely accounts for the bleeding phenotype in GSD Ia, elevated plasma corticosterone decreases the levels of proinflammatory monocytes. These studies highlight the necessity of maintaining good glycemic control in GSD Ia.

## Highlights

• Fasting-induced hypoglycemia in hepatocyte-specific *G6pc1* deficiency disturbs platelet aggregation.
• Fasting-induced hypoglycemia in hepatocyte-specific *G6pc1* deficiency decreases blood monocytes.
• Fasting-induced hypoglycemia in hepatocyte-specific *G6pc1* deficiency increases plasma corticosterone.

## List of abbreviations

GSD Ia: glycogen storage disease type 1a
G6PC1: glucose-6-phosphatase catalytic subunit
L-*G6pc*^−/−^: hepatocyte-specific *G6pc1* deficiency
G6P: glucose-6-phosphate
UCCS: uncooked cornstarch
NAFLD: non alcoholic fatty liver disease
ZT: zeitgeber time
WBC: white blood cell
RBC: red blood cell
CCR2: C–C chemokine receptor 2
CXCR4: C-X-C motif chemokine receptor 4
VLA4: very late antigen-4
LSK: Lin^-^Sca1^+^cKit^+^ cells
CMP: common myeloid progenitors
GMP: granulocyte/monocyte progenitors
MCP-1: monocyte chemoattractant protein-1
M-CSF: macrophage colony-stimulating factor
G-CSF: granulocyte colony-stimulating factor
FFA: free fatty acid
PNMT: phenylethanolamine *N*-methyltransferase
VLDL-TG: very-low-density lipoprotein triglycerides
LPL: lipoprotein lipase
vWF: von Willebrand factor
PT: prothrombin time
aPTT: activated partial thromboplastin time
SGLT2: sodium–glucose co-transporter 2
GLUT: glucose transporter
PAR4: protease-activated receptor 4 peptide

## 1 Introduction

Glycogen storage disease type 1a (GSD Ia) is an inborn error of carbohydrate metabolism with an incidence of 1 in 100,000 births [1]. GSD Ia is caused by mutations in the gene encoding the catalytic subunit of the glucose-6-phosphatase enzyme (G6PC1) [[2], [3], [4]]. *G6PC1* is expressed in the liver, kidney, and intestine, and catalyzes the hydrolysis of glucose-6-phosphate (G6P) to glucose [1]. Because of the essential role of *G6PC1* in endogenous glucose production, GSD Ia patients present with life-threatening fasting hypoglycemia when untreated [1,5]. To prevent hypoglycemia, patients receive dietary therapy that consists of frequent small doses of the slow release carbohydrate source uncooked cornstarch (UCCS) during the day, and UCCS or continuous gastric drip feeding during the night [6,7].

Despite intensive dietary treatment, short periods of hypoglycemia still occur in GSD Ia, generally referred to as poor glycemic control [6,7]. GSD Ia patients exhibit hyperlipidemia, which is aggravated by poor glycemic control [5,[8], [9], [10]]. In addition, they develop severe hepatomegaly and nonalcoholic fatty liver disease (NAFLD) as a consequence of excessive glycogen and lipid accumulation [1,11,12], and hepatocellular adenoma, which may progress to carcinoma [6,7,13,14]. Despite prominent hyperlipidemia, GSD Ia patients exhibit a 10% reduction in carotid intima-media thickness [15], reflecting decreased atherosclerotic lesions in the carotid arteries.

Under conditions of poor glycemic control, GSD Ia patients show increased bleeding tendency, which complicates surgical procedures [[16], [17], [18]]. This can be prevented by continuous gastric drip feeding to maintain euglycemia for 24 h before surgery [[16], [17], [18]]. Several pathways have been proposed to decrease atherosclerosis and enhance bleeding in GSD Ia [16,[18], [19], [20]]. The exact mechanisms remain elusive, likely due to the relatively small number of GSD Ia patients included in these studies and the high level of clinical heterogeneity [21]. Hypoglycemia caused by poor glycemic control could contribute to the low incidence of atherosclerosis in GSD Ia. Hypoglycemia may decrease blood monocytes, as suggested by others [23]. Low monocyte numbers may decrease atherosclerosis and monocyte-platelet aggregates, which induce coagulation [24,25]. Hypoglycemia may also affect platelet aggregation directly [18,26].

We investigated the effects of glycemic control in GSD Ia on leukocyte levels along with coagulation and the mechanisms involved. We used a mouse model of GSD Ia with hepatocyte-specific *G6pc1* deficiency (L*-G6pc*^*−/−*^ mice) that were studied under fed or fasted conditions, to match good or poor glycemic control in GSD Ia, respectively [10].

## 2 Materials and methods

### 2.1 Animals

Female *B6.G6pc1*^*lox/lox*^ and *B6.G6pc1*^*lox/lox*^*.SA*^*CreERT2*^ mice were housed in a light (lights on at 7:00 AM. *i.e.* Zeitgeber time (ZT) 0, lights off at 7:00 PM, *i.e.* ZT12) and temperature (21 °C) -controlled facility. Mice had free access to water and a standard chow diet (67% carbohydrates, consisting of the low glycemic index carbohydrate sources such as wheat (glycemic index 54), barley (glycemic index 28), and corn gluten, along with 23% protein and 10% fat (RMH-B, AB diets, Woerden, The Netherlands)). At 8–12 weeks of age, mice received intraperitoneal tamoxifen injections (T5648; Sigma–Aldrich, St. Louis, MO, USA) (1 mg/day in 95% sunflower oil/5% ethanol) for five consecutive days to generate hepatocyte-specific *G6pc1* deficient mice (L-*G6pc*^−/−^) and littermate floxed controls (control), as described previously [27]. Female littermates were randomly assigned to experimental groups and the number of mice used for each experiment is indicated in the figure legends. No inclusion or exclusion criteria were used. Experiments were performed for 6–16 weeks after tamoxifen injections at ZT1 (fed), ZT7 (6 h fast during the inactive period or nonfasted), and in case of fasting, the next morning at ZT1 (refeeding during the inactive and active period). All animal studies were approved by the Institutional Animal Care and Use Committee from the University of Groningen under permit number AVD105002015244 and adhered to guidelines set out in the 2010/63/EU directive.

### 2.2 Blood glucose levels

Blood glucose levels were measured using an Accu-Chek Performa glucose meter (Roche, Basel, Switzerland) and Accu-Chek Performa testing strips (06454011; Roche, Basel, Switzerland).

### 2.3 White blood cell and platelet counts

Blood samples were collected by tail bleeding into EDTA-coated tubes. Total white blood cell (WBC) and platelet counts were measured using the Medonic CD620 hematology analyzer (Boule Medical, Spanga, Sweden).

### 2.4 Flow cytometry

Blood samples were collected by tail bleeding into EDTA-coated tubes and kept on ice. For analysis of blood leukocyte subsets, samples were kept at 4 °C during the whole procedure, unless stated otherwise. Red blood cells (RBCs) were lysed for 5 min (BD Pharm Lyse; BD Bioscience, Franklin Lakes, NJ, USA) and WBCs were centrifuged, washed, and resuspended in HBSS (0.1% BSA and 0.5 mM EDTA). To assess monocytes, monocyte subsets, neutrophil, B-cell and T-cell populations, cells were stained with a cocktail of antibodies: CD45-APC-Cy7 (557659; BD Biosciences, Franklin Lakes, NJ, USA), CD115-APC (17-1152-82; eBioscience, San Diego, CA, USA), Ly6C/G-PercP-Cy5.5 (561103; BD Biosciences, Franklin Lakes, NJ, USA), TCRβ-PB (109226; Biolegend, San Diego, CA, USA), and CD19-PE (152408; Biolegend, San Diego, CA, USA) for 30 min on ice in the dark. Monocytes were identified as CD45^hi^CD115^hi^, and further separated into Ly6C^lo^ and Ly6C^hi^ subsets based on the marker Ly6C. Neutrophils were identified as CD45^hi^CD115^lo^Ly6G^hi^. Lymphocytes were identified as CD45^hi^CD115^lo^Ly6C^lo^/G^lo^, and further separated into B-cells identified as CD19^hi^, and T-cells identified as TCRβ^hi^. For measurements of monocyte and neutrophil surface marker expression, cells were stained with CD45-APC-Cy7 (557659; BD Biosciences, Franklin Lakes, NJ, USA), CD115-APC (17-1152-82; eBioscience, San Diego, CA, USA) or CD115-PE (135506; Biolegend, San Diego, CA, USA), and Ly6C/G-PercP-Cy5.5 (561103; BD Biosciences, Franklin Lakes, NJ, USA). C–C chemokine receptor 2 (CCR2) -PE (FAB5538P; R&D systems, Minneapolis, MN, USA), CD11b-PB (552093; BD Biosciences, Franklin Lakes, NJ, USA), CD62L-APC (17-0621-81; eBioscience, San Diego, CA, USA), C-X-C motif chemokine receptor 4 (CXCR4)-FITC (551967; BD Biosciences, Franklin Lakes, NJ, USA), CXCR2-APC (149604; Biolegend, San Diego, CA, USA), or very late antigen-4 (VLA4) -PE (553157; BD Biosciences, Franklin Lakes, NJ, USA) were added to this panel.

For analysis of bone marrow leukocyte and progenitor cell populations, the femur and tibia were collected, bone marrow (BM) was harvested and mashed through a 40 μm strainer. RBCs were lysed for 2 min on ice. WBCs were centrifuged, washed, and resuspended in HBSS (0.1% BSA and 0.5 mM EDTA). Total WBC counts were measured using the Medonic CD620 hematology analyzer (Boule Medical, Spanga, Sweden). Leukocyte subsets in BM were assessed by flow cytometry using the staining described for blood WBCs. For analysis of progenitor populations, cells were stained with a cocktail of antibodies (all from Biolegend, San Diego, CA, USA, unless stated otherwise): Sca1-BV421 (108127), cKit-APC (105812), CD127-PE (135010), CD16/32-PE-Cy7 (101318), CD34-FITC (553733; BD biosciences, Franklin Lakes, NJ, USA), B220-A700 (103231), CD11b-A700 (101222), CD3-A700 (100216), Ly6C/G-A700 (108422), and Ter119-A700 (116220) for 30 min on ice in the dark. Hematopoietic stem and progenitor cells were identified as CD127^-^, lineage (Lin)^-^, Sca1^+^, and cKit^+^ (LSK). Hematopoietic progenitors were identified as Lin^−^, CD127^-^, Sca-1^-^, cKit^+^ cells, and further separated into CD34^int^, CD16/32^int^, common myeloid progenitors (CMP), and CD34^int^, CD16/32^hi^ granulocyte/monocyte progenitors (GMP).

For analysis of hepatic monocytes and neutrophils, livers were collected, digested using collagenase (1.5 mg/ml in PBS) (C5138; Sigma–Aldrich, St. Louis, MO, USA) for 45 min at 37 °C and mashed through a 40 μm strainer. Up to 50 ml PBS was added and samples were centrifuged at 70×*g* with brake settings off for 5 min. The upper layer containing the hepatic leukocytes was centrifuged at 400×*g* for 10 min and RBCs were lysed for 2 min on ice. Samples were centrifuged, washed, and resuspended in HBSS (0.1% BSA and 0.5 mM EDTA). CD45^+^ cells were isolated from liver homogenates using CD45-coated magnetic beads (130-052-301; Miltenyi Biotec, Bergisch Gladbach, Germany) according to the manufacturer's instructions. Total WBC counts were measured using the Medonic CD620 hematology analyzer (Boule Medical, Spanga, Sweden). To assess hepatic monocytes and neutrophils, CD45^+^ cells were stained with CD45-APC-Cy7 (557659; BD Biosciences, Franklin Lakes, NJ, USA), CD11b-PB (552093; BD Biosciences, Franklin Lakes, NJ, USA), and Ly6C/G-PercP-Cy5.5 (561103; BD Biosciences, Franklin Lakes, NJ, USA) as described for blood WBCs. Monocytes were identified as CD45^hi^CD11b^hi^Ly6C^hi^SSC^lo^ and neutrophils as CD45^hi^CD11b^hi^Ly6G^hi^SSC^hi^.

All samples were analyzed on an LSRII (BD Biosciences, Franklin Lakes, NJ, USA), running FACSDiVa software (BD Biosciences, Franklin Lakes, NJ, USA). Data were analyzed using FlowJo software (FlowJo, Ashland, OR, USA). CCR2 expression was analyzed as mean fluorescence intensity (MFI).

### 2.5 ELISAs

Blood samples were collected. Plasma was separated by centrifugation, and monocyte chemoattractant protein-1 (MCP-1), macrophage-colony stimulating factor (M-CSF), and granulocyte-colony stimulating factor (G-CSF) were measured using ELISA kits (MJE00, MMC00, and MCS00, respectively; R&D systems, Minneapolis, MN, USA) according to the manufacturer's instructions.

### 2.6 Mifepristone treatment

Six weeks after tamoxifen injections, mice received an intraperitoneal injection with the glucocorticoid receptor antagonist mifepristone (M8046; Sigma–Aldrich, St. Louis, MO, USA) (25 mg/kg in 90% sunflower oil/10% ethanol) or vehicle at the start of the 6 h fasting period.

### 2.7 Plasma triglycerides and lipoprotein analysis

Blood samples were collected. Plasma was separated by centrifugation and plasma triglyceride (TG) and free fatty acid (FFA) levels were measured using enzymatic kits (157.109.910.917 and 157.819.910.935, respectively; Diasys Diagnostic Systems, Holzheim, Germany) with Precimat Glycerol or FFA standard FS (10,166,588; Roche, Mannheim, Germany and 157.809.910.065; Diasys Diagnostic Systems, respectively) for the calibration curve. Lipoprotein TG distribution was measured by fast performance liquid chromatography (FPLC) using a system containing a PU-4180 pump with a linear degasser and UV-4075 UV/VIS detectors (Jasco, Tokyo, Japan). Pooled plasma samples (n = 8–12) were injected onto a Superose 6 Increase 10/300 GL column (GE Healthcare, Hoevelaken, The Netherlands) and eluted at a constant flow rate of 0.31 ml/min in PBS (pH 7.4). Triglycerides were measured in line by the addition of TG reagent (157.109.910.917; Diasys, Holzheim, Germany) at a constant flow rate of 0.1 ml/min using an additional PU-4080i infusion pump (Jasco, Tokyo, Japan). Data acquisition and analysis were performed using ChromNav software (version 1.0; Jasco, Tokyo, Japan).

### 2.8 Flow cytometry of platelet-leukocyte aggregates

For the analysis of platelet-leukocyte aggregates, blood was collected by retro-orbital bleeding into tubes containing 3.2% sodium citrate (9:1 v/v) (41.1506.002; Sarstedt, Nümbrecht, Germany) and kept at RT. Samples were kept at 4 °C for all subsequent steps. RBCs were lysed for 5 min (BD Pharm Lyse, BD Bioscience, Franklin Lakes, NJ, USA), samples were centrifuged, washed, and resuspended in HBSS (0.1% BSA and 0.5 mM EDTA). To assess platelet-monocyte and platelet-neutrophil aggregation, samples were incubated with a cocktail of antibodies: CD45-APC-Cy7 (557659; BD Biosciences, Franklin Lakes, NJ, USA), CD115-PE (135506; Biolegend, San Diego, CA, USA), Ly6C/G-PercP-Cy5.5 (571103; BD Biosciences, Franklin Lakes, NJ, USA), CD41-FITC (133904; Biolegend, San Diego, CA, USA) for 30 min on ice in the dark. Platelet-monocyte aggregates were identified as CD45^hi^CD115^hi^CD41^hi^, and were further separated into platelet-Ly6C^lo^ monocyte aggregates, defined as CD45^hi^CD115^hi^Ly6C^lo^CD41^hi^, and platelet-Ly6C^hi^ monocyte aggregates, defined as CD45^hi^CD115^hi^Ly6C^hi^CD41^hi^. Platelet-neutrophil aggregates were identified as CD45^hi^CD115^lo^Ly6G^hi^CD41^hi^. All samples were analyzed on an LSRII (BD Biosciences, Franklin Lakes, NJ, USA), running FACSDiVa software (BD Biosciences, Franklin Lakes, NJ, USA). The data were analyzed using FlowJo software (FlowJo, Ashland, OR, USA).

### 2.9 Plasma corticosterone, epinephrine, and norepinephrine

Blood was collected by retro-orbital bleeding into EDTA-coated tubes (450532; Greiner Bio-One, Kremsmünster, Austria) and kept on ice. Plasma was separated by centrifugation and 5 mg reduced glutathione (G4251; Sigma–Aldrich, St. Louis, MO, USA) was added per mL of plasma after centrifugation to prevent degradation of epinephrine and norepinephrine. Plasma was snap-frozen and stored at −80 °C until further use. Corticosterone, epinephrine, and norepinephrine were measured by online solid phase extraction (SPE) in combination with isotope dilution liquid chromatography tandem mass spectrometry (LC-MS/MS). Corticosterone measurement was performed essentially as described by Hawley et al. [28], using corticosterone-D4 as a stable isotope-labeled internal standard on an online SPE manager in combination with a XEVO TQ mass spectrometer (Waters, Milford, MA, USA). Epinephrine and norepinephrine were analyzed as described by Van Faassen et al. [29], using epinephrine-D3 and norepinephrine-D6 as stable isotope-labeled internal standard on a Symbiosis Pharma online SPE system (Spark Holland, Emmen, The Netherlands) in combination with a Xevo TQ mass spectrometer (Waters, Milford, MA, USA).

### 2.10 Adrenal gland mass

Twelve weeks after tamoxifen injections, mice were sacrificed by cardiac puncture in the fed or fasted condition. Adrenal glands were collected, cleaned of surrounding fat, and weighed.

### 2.11 Tail vein bleeding assay

The tail vein bleeding assay was performed as described previously [30]. Briefly, mice were anesthetized with isoflurane. A 10 mm segment of the distal tip of the tail was removed using a scalpel. The tail was immediately immersed in a 50 ml tube containing 50 ml phosphate-buffered saline (PBS) prewarmed to 37 °C. Time until the bleeding ceased was recorded with a maximum bleeding time of 300 s. Volume of blood loss during the bleeding assay was quantified by measuring absorbance at 550 nm using a spectrophotometer (Genesys 10S UV-Vis; Thermofisher Scientific, Waltham, MA, USA).

### 2.12 Prothrombin time, activated partial prothrombin time, and plasma coagulation factors

Blood was collected by cardiac puncture into tubes containing 3.2% sodium citrate (9:1 v/v) (41.1506.002; Sarstedt, Nümbrecht, Germany). Samples were immediately centrifuged at RT to collect plasma. Plasma was stored at −80 °C until further use. Prothrombin time (PT) and activated partial thrombin time (aPTT) were assessed on an automated coagulation analyzer (CS-2100i) with reagents (PT-Innovin for PT and ActinFS for aPTT) and protocols from the manufacturer (Siemens, Marburg, Germany). Levels of factor V (FV), VII (FVII), and FVIII (FVIII) were determined on an automated coagulation analyzer (CS2100i) based on the one-stage clotting assay with factor-deficient plasma (Siemens, Marburg, Germany). Testing was performed in accordance with the protocols from the manufacturer (Siemens). Levels are denoted as percentages of standard human plasma (Siemens, Marburg, Germany).

### 2.13 Von Willebrand ristocetin cofactor

Blood was collected by cardiac puncture into tubes containing 3.2% sodium citrate (9:1 v/v) (41.1506.002; Sarstedt, Nümbrecht, Germany). Samples were immediately centrifuged at RT to collect plasma. Plasma was stored at −80 °C until use. Von Willebrand ristocetin cofactor (vWF RCF) levels were determined on a CS2100i coagulation analyzer with the Innovance vWF activity kit (Siemens, Marburg, Germany) in accordance with the protocol from the manufacturer with all equipment and reagents from Siemens (Marburg, Germany).

### 2.14 Platelet aggregation

To assess platelet aggregation, blood was drawn by cardiac puncture and collected into tubes containing 3.2% sodium citrate (9:1 v/v) (41.1506.002; Sarstedt, Nümbrecht, Germany). Samples were kept at RT during the whole procedure. Samples were centrifuged at 110×*g* and platelet-rich plasma (PRP) was collected. PRP was diluted to a final concentration of 200∗10^6^ platelets per mL using platelet-poor plasma (PPP) from the same mouse. Platelet aggregation in response to 10 μM ADP was measured using light transmission aggregometry (Chrono-log Model 700; Chrono-log Corporation, PA, USA) at 37 °C, stirring at 1200 rpm. Light transmission was recorded for 10 min using AggroLink 8 software.

### 2.15 Statistical analysis

All data are presented as mean ± SEM. The unpaired t-test was used to compare two datasets. To compare ≥3 groups, a one-way ANOVA with Bonferroni post hoc test was performed. Group size and statistical tests are reported in the figure legends. The criterion for significance was set at P < 0.05. Statistical analysis was performed using GraphPad Prism 5 (GraphPad Software; San Diego, CA, USA).

## 3 Results

### 3.1 Fasting decreases blood leukocytes in L*-G6pc*^−/−^ mice

**Figure 1:**
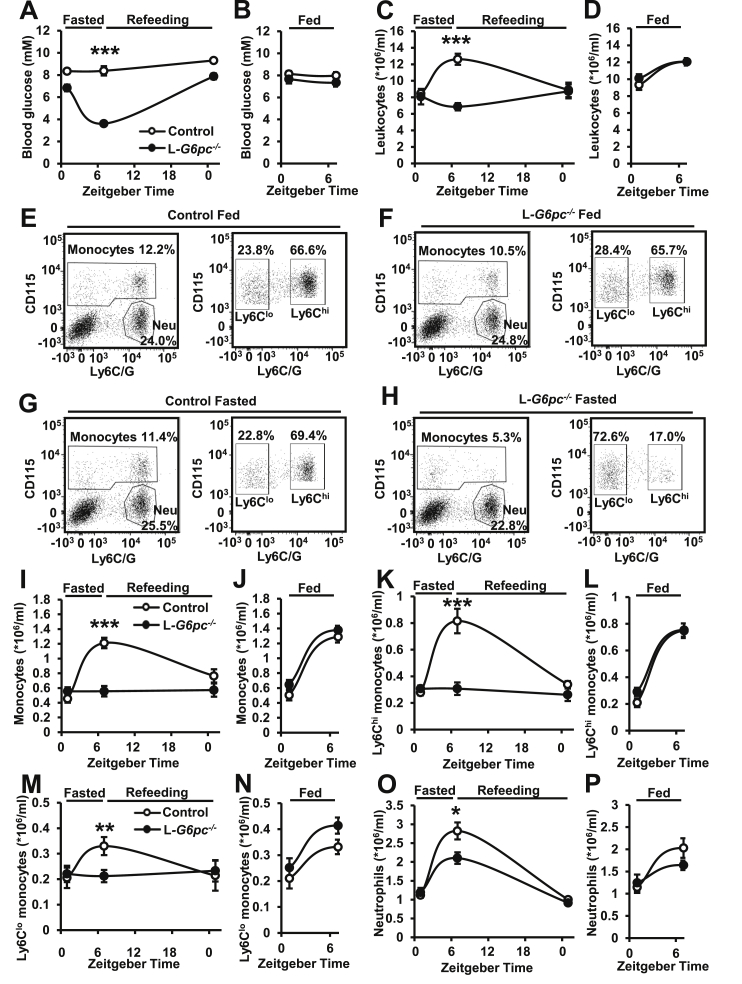
**Fasting decreases blood monocytes and neutrophils in L*-G6pc***^**−/−**^**mice.** Hepatocyte-specific *G6pc1* deficiency was induced by tamoxifen injections. After 6–16 weeks of tamoxifen injections, blood glucose and leukocyte levels were assessed at Zeitgeber time (ZT) 1 (fed), ZT7 (6 h fast during the inactive period or non fasted), and, in case of fasting, the next morning at ZT1 (refeeding during the inactive and active period). For each experiment, the same mouse was measured in the fed (ZT1) and fasted (ZT7) or non fasted (ZT7) condition, and in case of fasting, in the refed condition (ZT1, the next morning). (**A-B**) Blood glucose levels. (**C-D**) Total CD45^+^ leukocyte levels. (**E-H**) Representative flow cytometry plots of monocyte and neutrophils as a percentage of CD45^+^ leukocytes. Monocyte subsets are given in the fed (**E-F**) and fasted (**G-H**) condition. Neu denotes neutrophils. (**I–P**) Quantification of total monocytes, monocyte subsets, and neutrophils in the fed, fasted or non fasted, and refed condition. (n = 12–16). Data are shown as mean ± SEM. ∗*p* < 0.05, ∗∗*p* < 0.01, ∗∗∗*p* < 0.001 by t-test. Experiments have been repeated 6 times with similar results.

We first confirmed that similar to previous studies [27,31], hepatocyte-specific *G6pc1* deficiency induces hypoglycemia after a fasting period of 6 h. For this purpose, we measured blood glucose levels at Zeitgeber time (ZT) 1 (fed), ZT7 (6 h fast during the inactive period or nonfasted), and the next morning at ZT1 (refeeding during the inactive and active period). In accordance with previous studies [27], L*-G6pc*^−/−^ mice exhibited hypoglycemia (blood glucose ≤ 4.0 mM) after a 6 h fast, with average blood glucose levels of ~3.6 mM (Figure 1A). Refeeding restored blood glucose levels to ~7.9 mM (refeeding; ZT1) (Figure 1A). L*-G6pc*^−/−^ mice maintained euglycemia when they did not fast (Figure 1B) probably because of the chow diet given to GSD Ia patients that resembles the dietary therapy. GSD Ia patients are on a dietary therapy of uncooked cornstarch that has a glycemic index of 48 [32], and as a consequence induces the slow release of glucose, preventing hypoglycemia in these patients [7]. Chow diet contains 67% carbohydrates with a glycemic index of 54 and 28 (specified in the methods section), which explains why L*-G6pc*^−/−^ mice maintain euglycemia when not fasted. We observed the decrease in blood glucose levels in L*-G6pc*^−/−^ mice upon a 6 h fast at all time points measured throughout the study (Figure S1A and B).

Previous studies have revealed that prolonged fasting (20 h) leads to hypoglycemia, which decreases blood monocyte levels [23]. We thus assessed whether hepatocyte-specific *G6pc1* deficiency affected blood leukocytes in the fasted and nonfasted condition. Control mice exhibited an increase in total CD45^+^ blood leukocyte levels after fasting, because of circadian rhythmicity [33], whereas L*-G6pc*^−/−^ mice did not (Figure 1C). Refeeding restored blood leukocyte levels in L*-G6pc*^−/−^ mice (ZT1; refeeding) (Figure 1C). We observed no differences in blood leukocytes when L*-G6pc*^−/−^ mice were not fasted and they remained euglycemic (Figure 1D). Further analysis of leukocyte subsets in fasted animals denoted that hepatocyte-specific *G6pc1* deficiency decreased total monocytes by ~50%, mainly reflected by decreases in proinflammatory Ly6C^hi^ and to a lesser extent Ly6C^lo^ monocytes (Figure 1E–I, K, M). Refeeding restored monocyte levels (Figure 1I,K, M). Leukocyte levels were not different between the genotypes in the nonfasted condition (Figure 1J, L, N). We then inquired which fasting-induced changes could account for the decrease in blood Ly6C^hi^ monocytes in L*-G6pc*^−/−^ mice during fasting. Blood Ly6C^hi^ monocyte levels represented a strong correlation with blood glucose levels in L*-G6pc*^−/−^ mice, but not in control mice upon a 6 h fast (Fig. S2; Pearson correlation coefficient r = 0.65; p = 0.009), suggesting a major contribution of hypoglycemia to the decreased blood monocytes upon fasting. Fasting also increases plasma VLDL-TG and VLDL-cholesterol in L*-G6pc*^*−/−*^ mice [10]. However, elevated VLDL-cholesterol increases Ly6C^hi^ monocytes in mice [34]. We found that fasting did not affect plasma FFAs in L*-G6pc*^*−/−*^ mice, although plasma FFAs were elevated in the fed and fasted state, compared to controls (Fig. S3). Hence it is unlikely that lipids have contributed to the decreased blood Ly6C^hi^ monocytes in fasted L*-G6pc*^*−/−*^ mice. Though our data suggest that hypoglycemia is a key factor in decreasing blood monocytes, we cannot exclude that hormonal changes in fasted L*-G6pc*^*−/−*^ mice [35] may have contributed to the decrease in monocytes. Administration of glucose by gavage during the fasting period, as carried out in wild-type mice in experiments by Jordan et al. [23], led to an even further aggravated hypoglycemia in L*-G6pc*^*−/−*^ mice (results not shown), similar to rapid changes in blood glucose levels and rebound hypoglycemia that have been observed upon glucose administration in GSD Ia patients [7]. Therefore, we could not assess the effect of glucose on leukocyte changes in L*-G6pc*^*−/−*^ mice directly. Neutrophils exhibited a similar response to monocytes in fasted and refed L*-G6pc*^−/−^ mice, although fasting decreased neutrophils by only ~26% (Figure 1G,H,O,P). T- and B-cells were similarly affected (Figure S4A–F), with effects on B-cells being most pronounced.

Collectively, hepatocyte-specific *G6pc1* deficiency decreased circulating leukocytes upon a 6-h fast, which was restored by refeeding. All leukocyte populations were affected, and we observed major decreases in monocytes, particularly the Ly6C^hi^ population, along with B-cells.

### 3.2 Fasting increases plasma corticosterone levels in L*-G6pc*^−/−^ mice

We then sought to elucidate the mechanism causing the decrease in Ly6C^hi^ monocytes in L*-G6pc*^*−/−*^ mice upon fasting. MCP-1 enhances the recruitment of Ly6C^hi^ monocytes from bone marrow by interacting with CCR2 on these cells [[36], [37], [38]]. A previous study has revealed that hypoglycemia decreases blood monocytes as a consequence of a reduction in plasma MCP-1 levels [23]. MCP-1 levels in the plasma of L*-G6pc*^*−/−*^ mice were not affected compared to controls after a 6 h fast (Figure S5A). M-CSF was also not affected (Figure S5B). We observed a small increase in plasma G-CSF levels (Figure S5C), which cannot explain the fasting-induced reduction in blood neutrophils in L*-G6pc*^*−/−*^ mice. We then studied surface markers on monocytes and neutrophils. CCR2, CD11b, and VLA4 expression on Ly6C^hi^ monocytes were not affected in L*-G6pc*^*−/−*^ mice compared to controls after a 6 h fast (Figure S5D), as were CXCR4, CD62L, CXCR2, and CD11b on neutrophils (Figure S5E). This means hepatocyte-specific *G6pc1* deficiency does not affect surface markers on monocytes and neutrophils upon fasting, suggesting no difference in the functionality of these cells.

**Figure 2:**
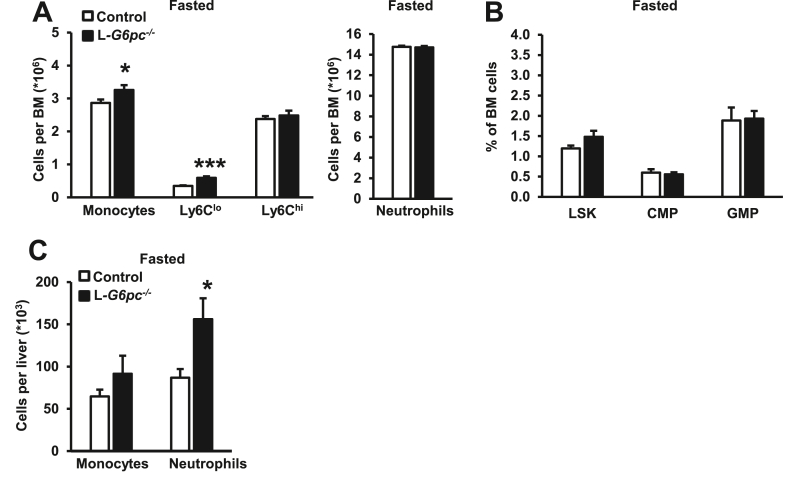
**Fasting increases bone marrow monocytes and hepatic neutrophils in L*-G6pc***^***−/−***^**mice.** Hepatocyte-specific *G6pc1* deficiency was induced by tamoxifen injections. Sixteen weeks after tamoxifen injections, (**A**) total monocytes, Ly6C^lo^ and Ly6C^hi^ monocyte subsets, and neutrophils in bone marrow (BM) were assessed by flow cytometry after a 6 h fast. (**B**) Lin^−^Sca1^+^cKit^+^ (LSK) cells, common myeloid progenitors (CMP), and granulocyte/monocyte progenitors (GMP) in the bone marrow were assessed by flow cytometry after a 6 h fast. (**C**) Total monocytes and neutrophils were assessed in the liver after a 6 h fast. Data are given as mean ± SEM. ∗*p* < 0.05, ∗∗∗*p* < 0.001 by t-test.

We then assessed where myeloid cells would accumulate during fasting-induced hypoglycemia in hepatocyte-specific *G6pc1* deficiency. We observed an increase in monocytes in bone marrow in L*-G6pc*^*−/−*^ mice upon fasting (Figure 2A). Although most bone marrow monocytes are Ly6C^hi^, the Ly6C^lo^ population was increased, perhaps because monocytes lose their Ly6C expression upon retention in the bone marrow. Stem and progenitor cells including LSK cells, CMPs, and GMPs that give rise to myeloid cells were not affected in the bone marrow of fasted L*-G6pc*^*−/−*^ mice (Figure 2B), or in the spleen or liver (results not given). Livers from L*-G6pc*^*−/−*^ mice exhibited increased neutrophils (Figure 2C), in accordance with a previous study where this was attributed to increased hepatic expression of the neutrophil chemoattractant keratinocyte-derived chemokine (KC) that stimulates neutrophil infiltration [39,40]. The decrease in blood monocytes upon fasting in L*-G6pc*^*−/−*^ mice may be caused by their accumulation in the bone marrow and the decrease in neutrophils because of their accumulation in the liver.

**Figure 3:**
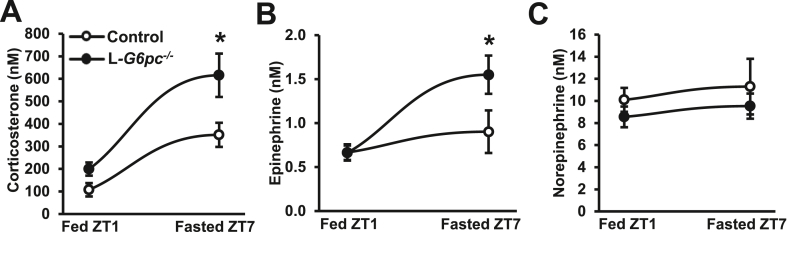
**Fasting increases plasma corticosterone and epinephrine levels in L*-G6pc***^***−/−***^**mice.** Hepatocyte-specific *G6pc1* deficiency was induced by tamoxifen injections. Fifteen weeks after tamoxifen injections, (**A**) plasma corticosterone, (**B**) epinephrine, and (**C**) norepinephrine levels were measured in the fed condition (ZT1) and again in the same mice after a 6 h fast (ZT7) using liquid chromatography-tandem mass spectrometry. (n = 11–14). Data are given as mean ± SEM. ∗*p* < 0.05 by t-test.

We further investigated the mechanism underlying the decrease in blood Ly6C^hi^ monocytes and their accumulation in bone marrow upon fasting-induced hypoglycemia in hepatocyte-specific *G6pc1* deficiency. Hypoglycemia evokes a stress response that leads to increased plasma corticosterone levels in mice [41]. Corticosterone impairs the recruitment of monocytes from the bone marrow [42,43]. Bone marrow monocytes are mostly from the Ly6C^hi^ subset [38]. We thus hypothesized that the decrease in Ly6C^hi^ monocytes was caused by increased corticosterone levels as a result of hypoglycemia in fasted L*-G6pc*^*−/−*^ mice. We measured levels of corticosterone and other stress hormones. While fasting increased plasma corticosterone levels in both genotypes, L*-G6pc*^−/−^ mice presented a 2-fold higher plasma corticosterone level than controls after fasting (Figure 3A). Adrenal gland mass was not affected after a 6 h fast, likely because the increase in plasma corticosterone was an acute effect (Figure S6). Corticosterone increases the activity of the enzyme phenylethanolamine N-methyltransferase (PNMT), which mediates the conversion of norepinephrine into epinephrine [44]. In line with an increase in corticosterone levels, epinephrine levels were also increased by 2-fold upon fasting in L*-G6pc*^−/−^ mice compared to controls (Figure 3B), whereas norepinephrine levels were unaffected (Figure 3C). Corticosterone and epinephrine have counter-regulatory effects on blood monocytes [42,43,45]. We observed a decrease in blood monocytes upon fasting and therefore hypothesized that the effect of corticosterone on blood monocytes is predominant, and investigated this further.

### 3.3 Inhibition of corticosterone signaling prevents the fasting-induced decrease in blood Ly6C^hi^ monocytes in L*-G6pc*^−/−^ mice

**Figure 4:**
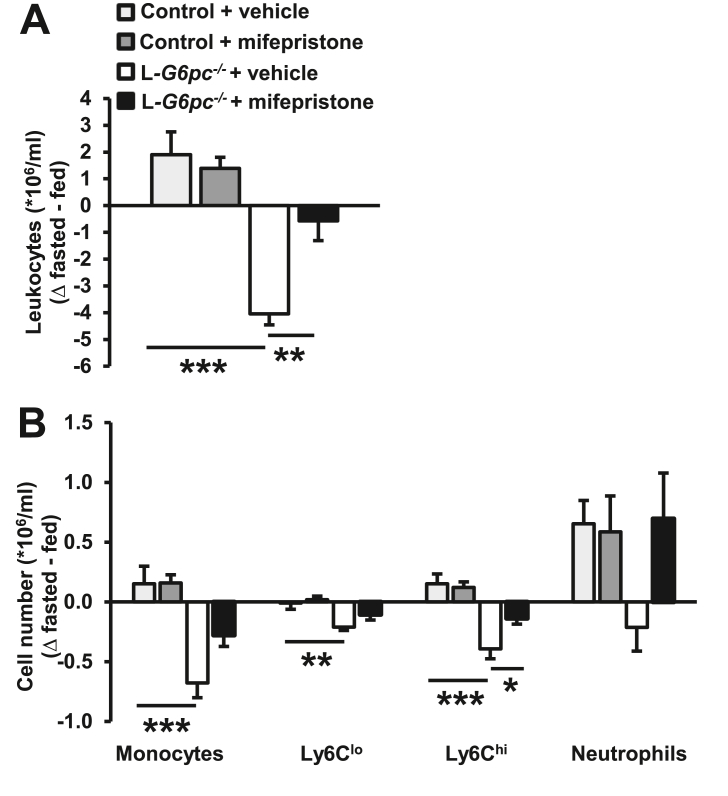
**Glucocorticoid receptor antagonist mifepristone prevents the fasting-induced decrease in blood Ly6C**^**hi**^**monocytes in L*-G6pc***^***−/−***^**mice.** Hepatocyte-specific *G6pc1* deficiency was induced by tamoxifen injections. Six weeks after tamoxifen injections, mice received an intraperitoneal injection with vehicle or mifepristone (25 mg/kg) at the start of the fasting period. (**A**) Total CD45^+^ leukocyte levels were assessed in the fed condition and again in the same mice after a 6 h fast. Difference in leukocyte levels after the 6 h fasting period is shown as Δ fasted – fed. (**B**) Quantification of total monocytes, monocyte subsets, and neutrophils in the fed and fasted condition, shown as Δ fasted – fed. (n = 8–12). Data are shown as mean ± SEM. ∗*p* < 0.05, ∗∗*p* < 0.01, ∗∗∗*p* < 0.001, by one-way ANOVA.

We then assessed whether the reduction in Ly6C^hi^ monocytes in fasted L*-G6pc*^−/−^ mice was dependent on the increase in plasma corticosterone levels. We injected mice with the glucocorticoid receptor antagonist mifepristone or vehicle at the start of the fasting period. Strikingly, mifepristone completely prevented the fasting-induced decrease in total CD45^+^ leukocytes in L*-G6pc*^*−/−*^ mice (Figure 4A). Mifepristone also prevented the fasting-induced decrease in Ly6C^hi^ monocytes in L*-G6pc*^−/−^ mice, whereas effects on other leukocyte populations did not reach statistical significance (Figures. 4B and S7). These data confirm our hypothesis that the decrease in Ly6C^hi^ monocytes in fasted L*-G6pc*^−/−^ mice is due to an exaggerated stress response caused by elevated plasma corticosterone.

A recent study has revealed that hypoglycemia in hepatocyte-specific *G6pc1* deficient mice impairs very-low-density lipoprotein (VLDL) catabolism, which was suggested to be caused by decreased lipoprotein lipase (LPL) activity [10]. As a result, plasma triglyceride levels were elevated [10]. Corticosterone may inhibit LPL activity [46,47]. We thus assessed whether the increase in plasma VLDL-triglycerides (VLDL-TG) in fasted L*-G6pc*^−/−^ mice was dependent on plasma corticosterone levels. Mifepristone prevented the increase in plasma VLDL-TG upon fasting in L*-G6pc*^−/−^ mice (Figure S8A and B), suggesting that elevated corticosterone levels contribute to fasting-induced hypertriglyceridemia in GSD Ia.

### 3.4 Fasting increases bleeding time in L*-G6pc*^−/−^ mice

**Figure 5:**
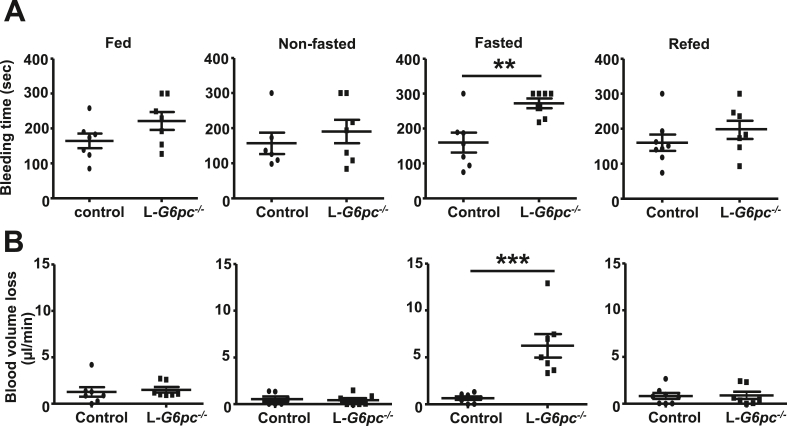
**Fasting increases bleeding time in L*-G6pc***^***−/−***^**mice.** Hepatocyte-specific *G6pc1* deficiency was induced by tamoxifen injections. After 6–12 weeks of tamoxifen injections, bleeding time was assessed in the fed, fasted, non fasted, and refed condition. (**A**) Bleeding time was recorded during a tail vein bleeding assay. (**B**) Blood volume loss per minute was quantified by measuring absorbance at 550 nm using a spectrophotometer. Each data point represents an individual mouse. (n = 6–8). Data are shown as mean ± SEM. ∗∗*p* < 0.01, ∗∗∗*p* < 0.001, by t-test. Experiments have been repeated 4 times with similar results.

The increased bleeding tendency in GSD Ia has been attributed to the decrease in von Willebrand factor (vWF) [19], disturbed platelet aggregation [16,18], and increased blood pressure [48]. Myeloid cells such as monocytes and neutrophils form leukocyte-platelet aggregates that contribute to coagulation [24]. Hence, a fasting-induced decrease in myeloid cells may reduce the formation of leukocyte-platelet aggregates, impair blood coagulation, and increase bleeding time. To test this hypothesis, we first assessed bleeding time in L*-G6pc*^−/−^ mice in the fed, fasted, nonfasted, and refed condition using the tail vein bleeding assay. Similar to observations in GSD Ia patients with poor glycemic control, fasting increased bleeding time in L*-G6pc*^−/−^ mice (Figure 5A). Further confirming these data, L*-G6pc*^−/−^ mice exhibited an increase in blood loss per minute upon fasting compared to controls (Figure 5B). The increased bleeding time and blood volume loss per minute were restored upon refeeding. Bleeding time and blood volume loss per minute were not affected in the fed and nonfasted states. Collectively, these data show that fasting prolongs bleeding time in L*-G6pc*^−/−^ mice.

We then investigated whether prolonged bleeding time upon fasting was caused by a decrease in monocyte-aggregates and neutrophil-aggregates because of a fasting-induced reduction in myeloid cells in L*-G6pc*^−/−^ mice. The percentage of monocytes and neutrophils that formed aggregates with platelets (CD41^+^) was not affected by hepatocyte-specific *G6pc1* deficiency (Figure S9A and B). This indicates that the capacity of monocytes and neutrophils to form aggregates with platelets did not change under fasting conditions. Nonetheless, the absolute decrease in these cell populations could contribute to less myeloid cell-platelet aggregates and increased bleeding during fasting in hepatocyte-specific *G6pc1* deficiency.

### 3.5 Fasting does not decrease coagulation factors in L*-G6pc*^−/−^ mice

**Figure 6:**
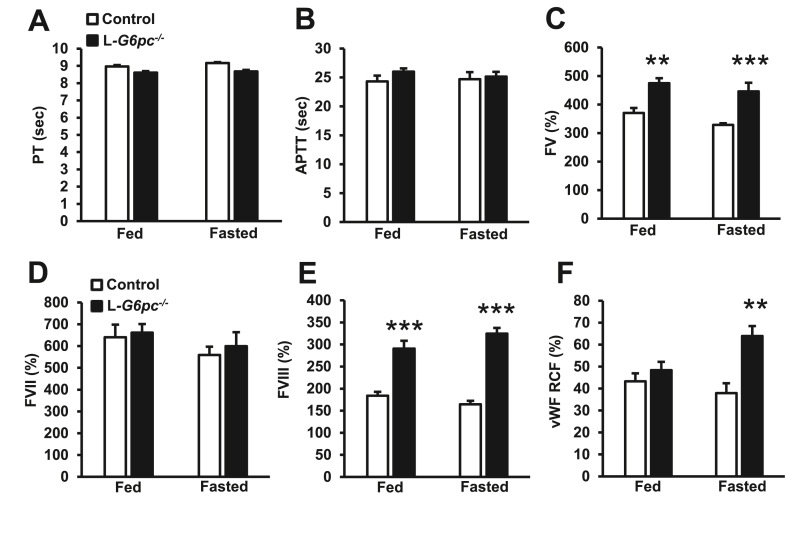
**Effects of hepatocyte-specific *G6pc1* deficiency on plasma levels of coagulation factors.** Hepatocyte-specific *G6pc1* deficiency was induced by tamoxifen injections. At 16 weeks after tamoxifen injections, (**A**) prothrombin time (PT) and (**B**) activated partial thromboplastin time (APTT) were assessed in the fed and fasted condition. (**C**) Plasma levels of coagulation factor V (FV), (**D**) factor VII (FVII), (**E**) factor VIII (FVIII), and (**F**) von Willebrand factor activity (vWF RCF) were measured in the fed and fasted condition. For each experimental condition (fed and 6 h fast), individual mice were used. (n = 6–8). Data are shown as mean ± SEM. ∗∗*p* < 0.01, ∗∗∗*p* < 0.001, by t-test.

We next investigated other mechanisms accounting for the fasting-induced increase in the bleeding time of L*-G6pc*^−/−^ mice. Since coagulation factors are mainly produced by the liver [49], where *G6pc1* is highly expressed [50], we first assessed whether coagulation factors secreted by the liver and downstream pathways were affected by hepatocyte-specific *G6pc1* deficiency. Prothrombin (PT) time, reflecting the intrinsic coagulation pathway that is dependent on factor VII and the common pathway consisting of factors X, II (thrombin), and I (fibrinogen), was not affected (Figure 6A). Activated partial thromboplastin time (aPTT), reflecting the extrinsic coagulation pathway that depends on factors XI, XII, VIII, and IX, was also not affected (Figure 6B). Plasma levels of coagulation factors V (FV) and VIII (FVIII) were increased in L*-G6pc*^−/−^ mice in the fed and fasted condition, while factor VII (FVII) was not affected (Figure 6C–E). The increases in FV and FVIII would increase coagulation, and hence, these increases are unlikely to explain the bleeding phenotype of L*-G6pc*^*−/−*^ mice. It has been described that GSD Ia patients exhibited decreased vWF levels [19]. On the contrary, we found that L*-G6pc*^−/−^ mice exhibited increased vWF levels upon fasting (Figure 6F). The increase in plasma vWF and FVIII could be the consequence of the fasting-induced increase in plasma corticosterone [[51], [52], [53]]. Similar to FV and FVIII, vWF would increase coagulation. Therefore, these increases cannot explain the prolonged bleeding phenotype in fasted L*-G6pc*^*−/−*^ mice. Hence, the bleeding phenotype in L*-G6pc*^−/−^ mice upon fasting is not due to decreases in vWF, FVIII, or coagulation factors produced by the liver.

### 3.6 Fasting disturbs platelet aggregation in L*-G6pc*^−/−^ mice

**Figure 7:**
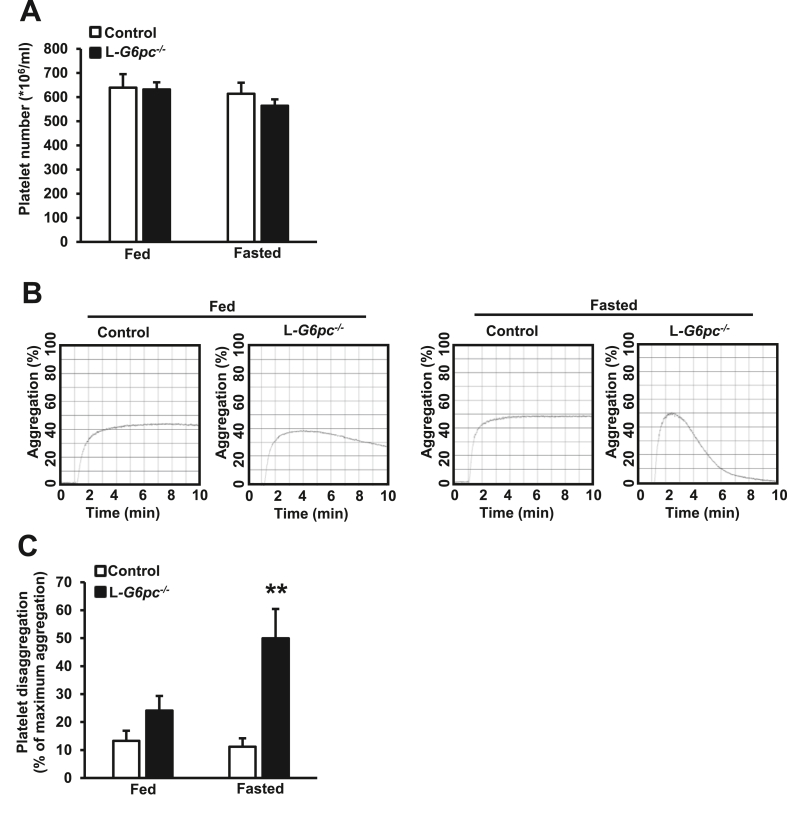
**Fasting induces platelet disaggregation in L*-G6pc***^***−/−***^**mice.** Hepatocyte-specific *G6pc1* deficiency was induced by tamoxifen injections. After 6–16 weeks of tamoxifen injections, blood platelet counts and platelet aggregation were assessed in the fed and fasted condition. (**A**) Blood platelet counts were measured in the fed condition and again in the same mouse after a 6 h fast. (n = 12–16). (**B–C**) Platelet aggregation in response to 10 μM ADP in platelet-rich plasma was measured using light transmission aggregometry. Representative platelet aggregation responses are shown (**B**) and quantified (**C**). Platelet disaggregation is denoted as the percentage of maximum aggregation response (**C**). For each experimental condition (fed and 6 h fast), individual mice were used. (n = 9–11). Data are shown as mean ± SEM. ∗∗*p* < 0.01, by t-test.

Even though the number of patients was limited (between 2 and 7 per study), it has been reported that GSD Ia patients exhibit disturbed platelet aggregation [[16], [17], [18]]. This could account for the prolonged bleeding time in GSD Ia. We thus assessed whether fasting affected platelet aggregation in L*-G6pc*^−/−^ mice. Blood platelet counts were not different between genotypes in both the fed and fasted condition (Figure 7A), but studies on platelet function did reveal distinct differences. Initially, ADP-induced stimulation of platelet-rich plasma from both controls and L*-G6pc*^−/−^ mice led to aggregation in both the fed and fasted condition (Figure 7B). Strikingly, after maximum aggregation had been reached, platelets isolated from fasted mice, but not from non-fasted L*-G6pc*^−/−^ mice, showed ~50% platelet disaggregation (Figure 7B,C). Hence, hepatocyte-specific *G6pc1* deficiency disturbs platelet aggregation after fasting. Among all mechanisms that we have explored, it is most likely that disturbed platelet aggregation accounts for the prolonged bleeding time in L*-G6pc*^−/−^ mice. We anticipate that this is also the main mechanism accounting for the bleeding tendency observed in hypoglycemic GSD Ia patients.

## 4 Discussion

Our findings reveal that hepatocyte-specific *G6pc1* deficiency in mice increases plasma corticosterone levels, decreases blood leukocytes, and disturbs platelet aggregation upon fasting. These results are most likely the consequence of fasting-induced hypoglycemia. The fasting-induced decrease in leukocytes in hepatocyte-specific *G6pc1* deficiency was mainly reflected by a decrease in Ly6C^hi^ monocytes and was prevented by the glucocorticoid receptor antagonist mifepristone. Furthermore, we found that hepatocyte-specific *G6pc1* deficiency prolonged bleeding time upon fasting due to disturbed platelet aggregation. Previous studies have recognized a relationship between hyperglycemia and monocytosis [54], and our data also indicate a similar relationship under conditions of fasting-induced hypoglycemia in hepatocyte-specific *G6pc1* deficiency, reflected by decrease in blood monocyte levels and disturbed platelet aggregation.

A relationship between glucose levels and blood monocytes in the setting of transient intermittent hyperglycemia independent of diabetes, and prolonged fasting has been shown previously [54]. Four injections of glucose over a period of 6 h increase blood monocyte levels, particularly the Ly6C^hi^ population in wild-type mice [54], and a fasting period of 20 h decreases blood monocytes in wild-type mice, with reversal by glucose administration [23]. Similarly, a 19 h fasting period in humans decreases plasma glucose levels and blood monocytes [23]. Several studies thus indicate a positive relationship between blood monocytes and plasma glucose levels [23,54], sharing similarities with our observations in a mouse model of hepatic GSD Ia.

While transient intermittent hyperglycemia increases blood monocytes because of elevated S100A8/A9 secretion by neutrophils as a consequence of glucose transporter (GLUT)-1 mediated glucose uptake [54], the decrease in blood monocytes upon hypoglycemia during prolonged fasting is considered to be secondary to a decrease in MCP-1 [23]. MCP-1 interacts with its receptor CCR2 on bone marrow monocytes to promote their egress from the bone marrow into blood [[36], [37], [38]]. We did not detect differences in plasma MCP-1 upon fasting-induced hypoglycemia in hepatocyte-specific *G6pc1* deficiency, but we did observe an increase in plasma corticosterone and epinephrine. Corticosterone decreases blood monocytes, but epinephrine has the opposite effect [45]. In diabetes, injections of insulin increase epinephrine levels [55] and blood monocytes [45], and presumably as a consequence thereof, patients with type 2 diabetes exhibit high levels of blood monocytes [22], although hyperglycemia also contributes to this effect [22]. Nonetheless, the question remains as to why monocytes were not increased as a consequence of the increased epinephrine in mice with hepatocyte-specific *G6pc1* deficiency, similar to effects of insulin injections in patients with diabetes. We assume that this relates to diabetic patients having impaired adrenocorticotrophic hormone responses to hypoglycemia, leading to the suppression of cortisol production by the adrenal gland [56]. As such, cortisol levels are not upregulated during hypoglycemia as a consequence of insulin injections [56], whereas epinephrine levels do increase, leading to elevated blood monocyte levels. Our studies showed that fasting-induced hypoglycemia in hepatocyte-specific *G6pc1* deficiency increases plasma levels of both corticosterone and epinephrine, and the decrease in blood monocytes suggests that the effect of corticosterone is predominant. Indeed, we found that the corticosterone receptor antagonist mifepristone reversed the fasting-induced decrease in Ly6C^hi^ monocytes in L-*G6pc*^−/−^ mice. We mainly observed differences in the Ly6C^hi^ monocyte subset, presumably because corticosterone impairs the egress of monocytes from the bone marrow [42,43,57], and most bone marrow monocytes are Ly6C^hi^ [38]. This is consistent with previous studies in rats demonstrating that acute stress or administration of corticosterone decreases blood monocytes within 2 h [42,58]. B-cells were also reduced by fasting in L*-G6pc*^−/−^ mice. Previous studies have shown that blood B-cell levels are controlled by corticosterone, at least when corticosterone levels are elevated for a long period (72 h) [59]. Mifepristone tended to reverse the decrease in B-cells in L*-G6pc*^*−/−*^ mice, although this did not reach statistical significance. Previous studies have also revealed that myocardial infarction (MI) leads to activation of the sympathetic nervous system, increasing norepinephrine production which acts on β_3_-adrenoceptors in bone marrow and promotes stem cell mobilization within 48 h of MI [60]. We did not observe any differences in norepinephrine levels or on hematopoietic stem and progenitor cells in the bone marrow, indicating that this mechanism did not play a role in L*-G6pc*^*−/−*^ mice.

GSD Ia patients display elevated levels of cortisol, the human homolog of corticosterone [61]. This was observed in the fed state, under conditions of good glycemic control. This effect was attributed to the production of NADPH in hepatocytes resulting from G6P accumulation. NADPH is a co-factor required for 11β-HSD1 reductase activity [62], resulting in the conversion of inactive glucocorticoids (cortisone in humans and 11-dehydrocortisone in mice) into active glucocorticoids (cortisol and corticosterone in humans and mice, respectively) [63]. GSD Ia patients exhibit increased conversion of administered cortisone into cortisol after overnight dexamethasone suppression [62], consistent with increased 11β-HSD1 reductase activity and elevated plasma cortisol levels [61]. Although L*-G6pc*^−/−^ mice do accumulate G6P in the liver, 11β-HSD1 reductase activity was not affected [62]. This is likely the consequence of 11β-HSD1 reductase activity being 20 times higher in mice than in humans [62,64], and may explain why plasma corticosterone is not elevated in L*-G6pc*^−/−^ mice in the fed state.

The increase in plasma cortisol in the fed state does not exclude that GSD Ia patients might exhibit high cortisol levels upon fasting-induced hypoglycemia, similar to mice with hepatocyte-specific *G6pc1* deficiency. Hypoglycemia in GSD Ia patients correlates with hyperlipidemia, in particular, increased VLDL-TGs [7,10,65,66]. Cortisol increases VLDL-TG plasma levels [67,68]. We found that the glucocorticoid receptor antagonist mifepristone suppressed the fasting-induced increase in plasma VLDL-TG in L*-G6pc*^*−/−*^ mice, consistent with a role for this antagonist in promoting VLDL-TG clearance, as suggested [69]. These data indicate that increased plasma cortisol caused by poor glycemic control contributes to high plasma VLDL-TG levels in GSD Ia patients [46,47].

Despite elevated VLDL-TG levels, a study in 9 GSD Ia patients has revealed that carotid intima-media thickness was not increased in GSD Ia patients compared to controls [15]. This effect can be explained as VLDL particles being too large to enter the vessel wall, similar to a setting of *Apolipoprotein C3* overexpression in mice [70]. Nonetheless, our data reveal that in a setting of poor glycemic control, levels of blood proinflammatory Ly6C^hi^ monocytes are decreased. Ly6C^hi^ monocytes infiltrate into atherosclerotic lesions [71]. Monocytosis enhances atherosclerosis, as revealed by studies in animal models [71] and humans [72]. The outcome of our studies suggests that conditions of poor glycemic control in GSD Ia patients may reduce the levels of proinflammatory monocytes. This could contribute to the decrease in atherosclerosis in GSD Ia.

We also found that fasted L*-G6pc*^*−/−*^ mice exhibit prolonged bleeding time in the tail vein bleeding assay, replicating the enhanced bleeding tendency in GSD Ia patients with poor glycemic control [[16], [17], [18]]. Various mechanisms have been proposed for this observation, including decreases in vWF [19], disturbed platelet aggregation [16,18], and increased blood pressure [48]. Because GSD Ia is a rare disorder, studies typically include small numbers of patients (between 2 and 7 per study), and hence, the bleeding phenotype in GSD Ia has not been evaluated systematically. We found that some coagulation factors secreted by the liver, one of the main organs affected in GSD Ia, were increased in L*-G6pc*^*−/−*^ mice upon fasting, as were FVIII and vWF; however, these increases would rather have led to increased coagulation and thus cannot explain the bleeding phenotype in GSD Ia. Strikingly, we observed that only in the fasted condition, L*-G6pc*^*−/−*^ mice showed disturbed platelet aggregation. This was reflected by ~50% platelet disaggregation in response to ADP after maximum aggregation had been reached. This indicates that the secondary wave of platelet aggregation, which is dependent on the release of ADP and ATP stored in the dense granules of platelets [73,74], is absent in hepatocyte-specific *G6pc1* deficiency. Therefore, the platelet activation process and recruitment of platelets cannot be amplified and a solid platelet plug cannot be formed [[73], [74], [75], [76]]. This is commonly observed in patients with dense granule storage pool disease [77], which is characterized by an almost complete absence of dense granule ADP and ATP [78,79] and a bleeding phenotype [79]. In accordance with these observations, it has been reported that platelets of 2 GSD Ia patients contained less ADP and ATP during hypoglycemia [18]. Continuous gastric drip feeding to maintain normoglycemia corrected ADP and ATP levels along with the disturbed platelet aggregation phenotype [18]. Together with our results in mice with hepatocyte-specific *G6pc1* deficiency, these findings indicate that disturbed platelet aggregation in GSD Ia is the consequence of low levels of ATP and ADP in the platelet dense granules due to hypoglycemia. In addition, we cannot exclude that the decrease in myeloid cells in L*-G6pc*^*−/−*^ mice may have led to a decrease in absolute numbers of monocyte- and neutrophil-platelet aggregates, which could contribute to increased bleeding in fasted L*-G6pc*^*−/−*^ mice compared to controls.

Several studies have shown that glucose levels affect platelet aggregation. Platelet deficiency of GLUT 1 and GLUT 3, which mediate glucose uptake, decreases platelet activation in response to protease-activated receptor 4 peptide (PAR4) and prolongs bleeding time in mice [80,81]. These observations support our finding that bleeding time is increased in fasting-induced hypoglycemia in L*-G6pc*^*−/−*^ mice. However, in contrast to findings in GSD Ia [[16], [17], [18]], acute hypoglycemia during an insulin stress test in humans increases platelet aggregation in response to ADP without affecting platelet ATP and ADP content [82]. The exact mechanism for this finding is unclear. Furthermore, acute insulin-induced hypoglycemia has been demonstrated to increase vWF levels in humans [83,84], likely the consequence of the hypoglycemia-induced increase in corticosterone levels [51,52] or endothelial dysfunction [84,85], and enhancing platelet aggregation. In sum, several mechanisms affecting platelet aggregation or disaggregation, including the one identified in GSD Ia, are regulated by blood glucose levels.

A limitation of our study is the use of adult, hepatocyte-specific *G6pc1* deficient mice. Whole body *G6pc1*^*−/−*^ mice require daily glucose injections to survive, they rarely live longer than 3 months, and cannot be used to investigate the effects of *G6pc1* deficiency during fasting [11,86,87]. Although kidney-specific and intestinal-specific *G6pc1* deficient mice do not become hypoglycemic upon fasting [[88], [89], [90]], we cannot exclude that in GSD Ia patients, loss of G6pc activity in kidney and intestine contributes to the bleeding phenotype under conditions of poor glycemic control.

## 5 Conclusion

We have shown that hepatocyte-specific *G6pc1* deficiency increases plasma corticosterone levels upon fasting, which decreases Ly6C^hi^ monocytes in the blood. Although direct effects of poor glycemic control on cortisol or leukocyte levels have not been evaluated in GSD Ia patients, this would be of interest, especially because increased plasma cortisol may contribute to elevated VLDL-TG under conditions of poor glycemic control in GSD Ia. In addition, we found that hepatocyte-specific *G6pc1* deficiency disturbs platelet aggregation during fasting-induced hypoglycemia, accounting for the bleeding phenotype in GSD Ia. Together, these results highlight the clinical necessity of maintaining good glycemic control in GSD Ia.

## Credit author statement

Anouk M. La Rose: Conceptualization, Methodology, Investigation, Writing – Original Draft preparation. Venetia Bazioti, Joanne A. Hoogerland, Arthur F. Svendsen, Anouk G. Groenen, Martijn van Faassen, Martijn G.S. Rutten, Niels J. Kloosterhuis, Bertien Dethmers-Ausema, J. Hendrik Nijland: Investigation. G. Mithieux, F. Rajas: Resources, Writing – Review & Editing. M.V. Lukens, F. Kuipers, O. Soehnlein, M.H. Oosterveer: Conceptualization, Writing – Review & Editing. M. Westerterp: Conceptualization, Methodology, Investigation, Writing – Review & Editing, Supervision, Funding Acquisition.

## References

[1] Chou J.Y., Jun H.S., Mansfield B.C. (2015). Type I glycogen storage diseases: disorders of the glucose-6-phosphatase/glucose-6-phosphate transporter complexes. Journal of Inherited Metabolic Disease.

[2] Lei K.J., Shelly L.L., Pan C.J., Sidbury J.B., Chou J.Y. (1993). Mutations in the glucose-6-phosphatase gene that cause glycogen storage disease type 1a. Science.

[3] Lei K.J., Chen Y.T., Chen H., Wong L.J.C., Liu J.L., McConkie-Rosell A. (1995). Genetic basis of glycogen storage disease type 1a: prevalent mutations at the glucose-6-phosphatase locus. The American Journal of Human Genetics.

[4] Chevalier-Porst F., Bozon D., Bonardot A.M., Bruni N., Mithieux G., Mathieu M. (1996). Mutation analysis in 24 French patients with glycogen storage disease type la. Journal of Medical Genetics.

[5] Chou J.Y., Kim G.-Y., Cho J.-H. (2017). Recent development and gene therapy for glycogen storage disease type Ia. Liver Research.

[6] Dambska M., Labrador E.B., Kuo C.L., Weinstein D.A. (2017). Prevention of complications in glycogen storage disease type Ia with optimization of metabolic control. Pediatric Diabetes.

[7] Rake J.P., Visser G., Labrune P., Leonard J.V., Ullrich K., Smit G.P.A. (2002). Guidelines for management of glycogen storage disease type I - European study on glycogen storage disease type I (ESGSD I). European Journal of Pediatrics.

[8] Carvalho P.M.S., Silva N.J.M.M., Dias P.G.D., Porto J.F.C., Santos L.C., Costa J.M.N. (2013). Glycogen Storage Disease type 1a - a secondary cause for hyperlipidemia: report of five cases. Journal of Diabetes and Metabolic Disorders.

[9] Rake J.P., Visser G., Labrune P., Leonard J.V., Ullrich K., Smit G.P. (2002). Glycogen storage disease type I : diagnosis, management, clinical course and outcome. Results of the European Study on Glycogen Storage Disease Type I ( ESGSD I ). European Journal of Pediatrics.

[10] Hoogerland J.A., Peeks F., Hijmans B., Wolters J.C., Kooijman S., Bos T. (2021). Impaired VLDL catabolism links hypoglycemia to hypertriglyceridemia in GSDIa. Journal of Inherited Metabolic Disease.

[11] Lei K., Chen H., Pan C., Ward J.M., Mosinger B., Lee E.J. (1996). Glucose-6-phosphatase dependent substrate transport in the glycogen storage disease type-1a mouse. Nature Genetics.

[12] Lei Y., Hoogerland J.A., Bloks V.W., Bos T., Bleeker A., Wolters H. (2020). Hepatic carbohydrate response element binding protein activation limits nonalcoholic fatty liver disease development in a mouse model for glycogen storage disease type 1a. Hepatology.

[13] Wang D., Fiske L., Carreras C., Weinstein D. (2011). Natural history of hepatorcellular adenoma formation in glycogen storage disease type I. Journal of Pediatrics.

[14] Beegle R.D., Brown L.M., Weinstein D.A. (2015). Regression of hepatocellular adenomas with strict dietary therapy in patients with glycogen storage disease type I. JIMD Report.

[15] Ubels F., Rake J., Smit P., Smit A., Slaets J. (2002). Is glycogen storage disease 1a associated with atherosclerosis?. European Journal of Pediatrics.

[16] Czapek E.E., Deykin D., Salzman E.W. (1973). Platelet dysfunction in glycogen storage disease type I. Blood.

[17] Corby D.G., Putnam C.W., Greene H.L. (1974). Impaired platelet function in glucose-6-phosphatase deficiency. Journal of Pediatrics.

[18] Hutton R.A., Macnab A.J., Rivers R.P.A. (1976). Defect of platelet function associated with chronic hypoglycaemia. Archives of Disease in Childhood.

[19] Mühlhausen C., Schneppenheim R., Budde U., Merkel M., Muschol N., Ullrich K. (2005). Decreased plasma concentration of von Willebrand factor antigen (VWF:Ag) in patients with glycogen storage disease type Ia. Journal of Inherited Metabolic Disease.

[20] Bandsma R.H.J., Rake J.P., Visser G., Neese R.A., Hellerstein M.K., Van Duyvenvoorde W. (2002). Increased lipogenesis and resistance of lipoproteins to oxidative modification in two patients with glycogen storage disease type 1a. Journal of Pediatrics.

[21] Peeks F., Steunenberg T.A.H., de Boer F., Rubio-gozalbo M.E., Williams M., Burghard R. (2017). Clinical and biochemical heterogeneity between patients with glycogen storage disease type IA : the added value of CUSUM for metabolic control. Journal of Inherited Metabolic Disease.

[22] Nagareddy P.R., Murphy A.J., Stirzaker R.A., Hu Y., Yu S., Miller R.G. (2013). Hyperglycemia promotes myelopoiesis and impairs the resolution of atherosclerosis. Cell Metabolism.

[23] Jordan S., Tung N., Casanova-Acebes M., Chang C., Cantoni C., Zhang D. (2019). Dietary intake regulates the circulating inflammatory monocyte pool. Cell.

[24] Mansour A., Roussel M., Gaussem P., Nédelec-Gac F., Pontis A., Flécher E. (2020). Platelet functions during extracorporeal membrane oxygenation. Platelet–Leukocyte aggregates analyzed by flow cytometry as a promising tool to monitor platelet activation. Journal of Clinical Medicine.

[25] Celi A., Pellegrini G., Lorenzet R., De Blasi A., Ready N., Furie B.C. (1994). P-selectin induces the expression of tissue factor on monocytes. Proceedings of the National Academy of Sciences of the U S A.

[26] Kraakman M.J., Lee M.K.S., Al-Sharea A., Dragoljevic D., Barrett T.J., Montenont E. (2017). Neutrophil-derived S100 calcium-binding proteins A8/A9 promote reticulated thrombocytosis and atherogenesis in diabetes. Journal of Clinical Investigation.

[27] Mutel E., Abdul-Wahed A., Ramamonjisoa N., Stefanutti A., Houberdon I., Cavassila S. (2011). Targeted deletion of liver glucose-6 phosphatase mimics glycogen storage disease type 1a including development of multiple adenomas. Journal of Hepatology.

[28] Hawley J.M., Owen L.J., Mackenzie F., Mussell C., Cowen S., Keevil B.G. (2016). Candidate reference measurement procedure for the quantification of total serum cortisol with LC-MS/MS. Clinical Chemistry.

[29] van Faassen M., Bischoff R., Eijkelenkamp K., de Jong W.H.A., van der Ley C.P., Kema I.P. (2020). Matrix derivatization combined with LC-MS/MS results in ultrasensitive quantification of plasma free metanephrines and catecholamines. Analytical Chemistry.

[30] Liu Y., Jennings N.L., Dart A.M., Du X.-J. (2012). Standardizing a simpler, more sensitive and accurate tail bleeding assay in mice. World Journal of Experimental Medicine.

[31] Hoogerland J.A., Lei Y., Wolters J.C., de Boer J.F., Bos T., Bleeker A. (2019). Glucose-6-Phosphate regulates hepatic bile acid synthesis in mice. Hepatology.

[32] Romano A., Mackie A., Farina F., Aponte M., Sarghini F., Masi P. (2016). Characterisation, in vitro digestibility and expected glycemic index of commercial starches as uncooked ingredients. Journal of Food Science & Technology.

[33] Casanova-Acebes M., Pitaval C., Weiss L.A., Nombela-Arrieta C., Chèvre R., A-González N. (2013). Rhythmic modulation of the hematopoietic niche through neutrophil clearance. Cell.

[34] Murphy A.J., Akhtari M., Tolani S., Pagler T., Bijl N., Kuo C. (2011). ApoE regulates hematopoeitic stem cell proliferation, monocytosis, and monocyte accumulation in atherosclerotic lesions in mice. Journal of Clinical Investigation.

[35] Jensen T.L., Kiersgaard M.K., Sørensen D.B., Mikkelsen L.F. (2013). Fasting of mice: a review. Laboratory Animals.

[36] Serbina N.V., Pamer E.G. (2006). Monocyte emigration from bone marrow during bacterial infection requires signals mediated by chemokine receptor CCR2. Nature Immunology.

[37] Combadière C., Potteaux S., Rodero M., Simon T., Pezard A., Esposito B. (2008). Combined inhibition of CCL2, CX3CR1, and CCR5 abrogates Ly6Chi and Ly6Clo monocytosis and almost abolishes atherosclerosis in hypercholesterolemic mice. Circulation.

[38] Tsou C.-L., Peters W., Si Y., Slaymaker S., Aslanian A.M., Weisberg S.P. (2007). Critical roles for CCR2 and MCP-3 in monocyte mobilization from bone marrow and recruitment to inflammatory sites. Journal of Clinical Investigation.

[39] Kim S.Y., Chen L.Y., Yiu W.H., Weinstein D.A., Chou J.Y. (2007). Neutrophilia and elevated serum cytokines are implicated in glycogen storage disease type Ia. FEBS Letters.

[40] Kim S.Y., Weinstein D.A., Starost M.F., Mansfield B.C., Chou J.Y. (2008). Necrotic foci, elevated chemokines and infiltrating neutrophils in the liver of glycogen storage disease type Ia. Journal of Hepatology.

[41] Shum K., Inouye K., Chan O., Mathoo J., Bilinski D., Matthews S.G. (2001). Effects of antecedent hypoglycemia, hyperinsulinemia, and excess corticosterone on hypoglycemic counterregulation. American Journal of Physiology. Endocrinology and Metabolism.

[42] Dhabhar F.S., Miller A.H., McEwen B.S., Spencer R.L. (1995). Effects of stress on immune cell distribution: dynamics and hormonal mechanisms. The Journal of Immunology.

[43] Thompson J., van Furth R. (1973). The effect of glucocorticosteroids on the proliferation and kinetics of promonocytes and monocytes of the bone marrow. Journal of Experimental Medicine.

[44] Wurtman R.J., Axelrod J. (1965). Adrenaline synthesis: control by the pituitary gland and adrenal glucocorticoids. Science.

[45] Dimitrov S., Lange T., Born J. (2010). Selective mobilization of cytotoxic leukocytes by epinephrine. The Journal of Immunology.

[46] Koliwad S.K., Kuo T., Shipp L.E., Gray N.E., Backhed F., So A.Y.L. (2009). Angiopoietin-like 4 (ANGPTL4, fasting-induced adipose factor) is a direct glucocorticoid receptor target and participates in glucocorticoid-regulated triglyceride metabolism. Journal of Biological Chemistry.

[47] Dijk W., Kersten S. (2014). Regulation of lipoprotein lipase by Angptl4. Trends in Endocrinology and Metabolism.

[48] Yetman R.J., Andrew-Casal M., Hermida R.C., Dominguez B.W., Portman R.J., Northrup H. (2002). Circadian pattern of blood pressure, heart rate, and double product in liver glycogen storage disease. Chronobiology International.

[49] Kujovich J.L. (2015). Coagulopathy in liver disease: a balancing act. Hematology American Society Hematology Education Programs.

[50] Gjorgjieva M., Mithieux G., Rajas F. (2019). Hepatic stress associated with pathologies characterized by disturbed glucose production. Cell Stress.

[51] Majoor C.J., Sneeboer M.M.S., de Kievit A., Meijers J.C.M., van der Poll T., Lutter R. (2016). The influence of corticosteroids on hemostasis in healthy subjects. Journal of Thrombosis and Haemostasis.

[52] Von Känel R., Dimsdale J.E. (2000). Effects of sympathetic activation by adrenergic infusions on hemostasis in vivo. European Journal of Haematology.

[53] Dal Bo Zanon R., Fornasiero L., Boscaro M., Cappellato G., Fabris F., Girolami A. (1982). Increased factor VIII associated activities in Cushing's syndrome: a probable hypercoagulable state. Thrombosis & Haemostasis.

[54] Flynn M.C., Kraakman M.J., Tikellis C., Lee M.K.S., Hanssen N.M.J., Kammoun H.L. (2020). Transient intermittent hyperglycemia accelerates atherosclerosis by promoting myelopoiesis. Circulation Research.

[55] Jin W.L., Azuma K., Mita T., Goto H., Kanazawa A., Shimizu T. (2011). Repetitive hypoglycaemia increases serum adrenaline and induces monocyte adhesion to the endothelium in rat thoracic aorta. Diabetologia.

[56] Rhyu Y.A., Jang J.Y., Park S., An J.H., Kim D.L., Kim S.K. (2019). Impaired cortisol and growth hormone counterregulatory responses among severe hypoglycemic patients with type 2 diabetes mellitus. Endocrinology and Metabolism.

[57] Thompson J., van Furth R. (1970). The effect of glucocorticosteroids on the kinetics of mononuclear phagocytes. Journal of Experimental Medicine.

[58] Dhabhar F.S., Malarkey W.B., Neri E., McEwen B.S. (2012). Stress-induced redistribution of immune cells-From barracks to boulevards to battlefields: a tale of three hormones. Psychoneuroendocrinology.

[59] Courties G., Frodermann V., Honold L., Zheng Y., Herisson F.E., Schloss M.J. (2019). Glucocorticoids regulate bone marrow B lymphopoiesis after stroke. Circulation Research.

[60] Dutta P., Courties G., Wei Y., Leuschner F., Gorbatov R., Robbins C.S. (2012). Myocardial infarction accelerates atherosclerosis. Nature.

[61] Rossi A., Simeoli C., Salerno M., Ferrigno R., Della Casa R., Colao A. (2020). Imbalanced cortisol concentrations in glycogen storage disease type I: evidence for a possible link between endocrine regulation and metabolic derangement. Orphanet Journal of Rare Diseases.

[62] Walker E.A., Ahmed A., Lavery G.G., Tomlinson J.W., So Y.K., Cooper M.S. (2007). 11β-Hydroxysteroid dehydrogenase type 1 regulation by intracellular glucose 6-phosphate provides evidence for a novel link between glucose metabolism and hypothalamo-pituitary-adrenal axis function. Journal of Biological Chemistry.

[63] Tomlinson J.W., Walker E.A., Bujalska I.J., Draper N., Lavery G.G., Cooper M.S. (2004). 11β-Hydroxysteroid dehydrogenase type 1: a tissue-specific regulator of glucocorticoid response. Endocrine Reviews.

[64] Lavery G.G., Walker E.A., Draper N., Jeyasuria P., Marcos J., Shackleton C.H.L. (2006). Hexose-6-phosphate dehydrogenase knock-out mice lack 11β- hydroxysteroid dehydrogenase type 1-mediated glucocorticoid generation. Journal of Biological Chemistry.

[65] Derks T.G.J., van Rijn M. (2015). Lipids in hepatic glycogen storage diseases: pathophysiology, monitoring of dietary management and future directions. Journal of Inherited Metabolic Disease.

[66] Kishnani P.S., Austin S.L., Abdenur J.E., Arn P., Bali D.S., Boney A. (2014). Diagnosis and management of glycogen storage disease type I: a practice guideline of the American College of Medical Genetics and Genomics. Genetics in Medicine.

[67] Klausner H., Heimberg M. (1967). Effect of adrenalcortical hormones on release of triglycerides and glucose by liver. American Journal of Physiology.

[68] Arnaldi G., Scandali V.M., Trementino L., Cardinaletti M., Appolloni G., Boscaro M. (2010). Pathophysiology of dyslipidemia in Cushing's syndrome. Neuroendocrinology.

[69] Kroon J., Koorneef L.L., Van Den Heuvel J.K., Verzijl C.R.C., Van De Velde N.M., Mol I.M. (2018). Selective glucocorticoid receptor antagonist CORT125281 activates brown adipose tissue and alters lipid distribution in male mice. Endocrinology.

[70] Ebara T., Ramakrishnan R., Steiner G., Shachter N.S. (1997). Chylomicronemia due to Apolipoprotein CIII overexpression in Apolipoprotein E-null mice Apolipoprotein CIII – induced hypertriglyceridemia is not mediated by effects on Apolipoprotein E. Journal of Clinical Investigation.

[71] Swirski F.K., Libby P., Aikawa E., Alcaide P., Luscinskas F.W., Weissleder R. (2007). Ly-6Chi monocytes dominate hypercholesterolemia-associated monocytosis and give rise to macrophages in atheromata. Journal of Clinical Investigation.

[72] Coller B.S. (2005). Leukocytosis and ischemic vascular disease morbidity and mortality is it time to Intervene?. ATVB.

[73] Erhardt J.A., Pillarisetti K.T.J. (2003). Potentiation of platelet activation through the stimulation of P2X 1 receptors. Journal of Thrombosis and Haemostasis.

[74] Hoylaerts M.F., Oury C., Toth-zsamboki E., Vermylen J., Hoylaerts M.F., Toth-zsamboki E. (2000). ADP receptors in platelet activation and aggregation ADP receptors in platelet activation and aggregation. Platelets.

[75] Daniel J.L., Dangelmaier C., Jin J., Ashby B., Smith J.B., Kunapuli S.P. (1998). Molecular basis for ADP-induced platelet activation. Journal of Biological Chemistry.

[76] Jiang L., Xu C., Yu S., Liu P., Luo D., Zhou Q. (2013). A critical role of thrombin/PAR-1 in ADP-induced platelet secretion and the second wave of aggregation. Journal of Thrombosis and Haemostasis.

[77] Weiss H.J.L.B. (1988). The response of platelets to epinephrine in storage pool deficiency - evidence pertaining to the role of ADP in mediating primary and secondary aggregation. Blood.

[78] Dupuis A., Bordet J., Eckly A., Gachet C. (2020). Platelet δ -storage pool Disease : an update. Journal of Clinical Medicine.

[79] Pareti F.I., Day H.J., Mills D.C.B. (1974). Nucleotide and serotonin metabolism in platelets with defective secondary aggregation. Blood.

[80] Fidler T.P., Campbell R.A., Funari T., Chaudhuri D., Weyrich A.S., Abel E.D. (2017). Deletion of GLUT1 and GLUT3 reveals multiple roles for glucose metabolism in platelet and megakaryocyte function article deletion of GLUT1 and GLUT3 reveals multiple roles for glucose metabolism in platelet and megakaryocyte function. Cell Reports.

[81] Fidler T.P., Marti A., Gerth K., Middleton E.A., Campbell R.A., Rondina M.T. (2019). Glucose metabolism is required for platelet hyperactivation in a murine model of type 1 diabetes. Diabetes.

[82] Hutton R.A., Mikhailidis D., Dormandy K.M., Ginsburg J. (1979). Platelet aggregation studies during transient hypoglycaemia. A potential method for evaluating platelet function. Journal of Clinical Pathology.

[83] Fisher B.M., Quin J.D., Rumley A., Lennie S.E., Small M., MacCuish A.C.L.G. (1991). Effects of acute insulin-induced hypoglycaemia on haemostasis, fibrinolysis and haemorheology in insulin-dependent diabetic patients and control subjects. Clinical Science.

[84] Wright R.J., Newby D.E., Stirling D., Ludlam C.A., Macdonald I.A., Frier B.M. (2010). Effects of acute insulin-induced hypoglycemia on indices of inflammation. Diabetes Care.

[85] Lip G.Y.H., Blann A. (1997). von Willebrand factor: a marker of endothelial dysfunction in vascular disorders?. Cardiovascular Research.

[86] Yiu W.H., Lee Y.M., Peng W.T., Pan C.J., Mead P.A., Mansfield B.C. (2010). Complete normalization of hepatic G6PC deficiency in murine glycogen storage disease type Ia using gene therapy. Molecular Therapy.

[87] Salganik S.V., Weinstein D.A., Shupe T.D., Salganik M., Pintilie D.G., Petersen B.E. (2009). A detailed characterization of the adult mouse model of glycogen storage disease Ia. Laboratory Investigation.

[88] Clar J., Gri B., Calderaro J., Birling M.C., Hérault Y., Smit G.P.A. (2014). Targeted deletion of kidney glucose-6 phosphatase leads to nephropathy. Kidney International.

[89] Soty M., Penhoat A., Amigo-Correig M., Vinera J., Sardella A., Vullin-Bouilloux F. (2015). A gut-brain neural circuit controlled by intestinal gluconeogenesis is crucial in metabolic health. Molecular Metabolism.

[90] Penhoat A., Fayard L., Stefanutti A., Mithieux G., Rajas F. (2014). Intestinal gluconeogenesis is crucial to maintain a physiological fasting glycemia in the absence of hepatic glucose production in mice. Metabolism.

