# Supplementary material for: Hepatocyte-specific glucose-6-phosphatase deficiency disturbs platelet aggregation and decreases blood monocytes upon fasting-induced hypoglycemia
Source: Mol Metab. 2021 Jun 4;53:101265. doi: 10.1016/j.molmet.2021.101265 (PMC8243524; doi:10.1016/j.molmet.2021.101265)
Supplement: Multimedia component 1 [file mmc1.pptx]

## Slide 1
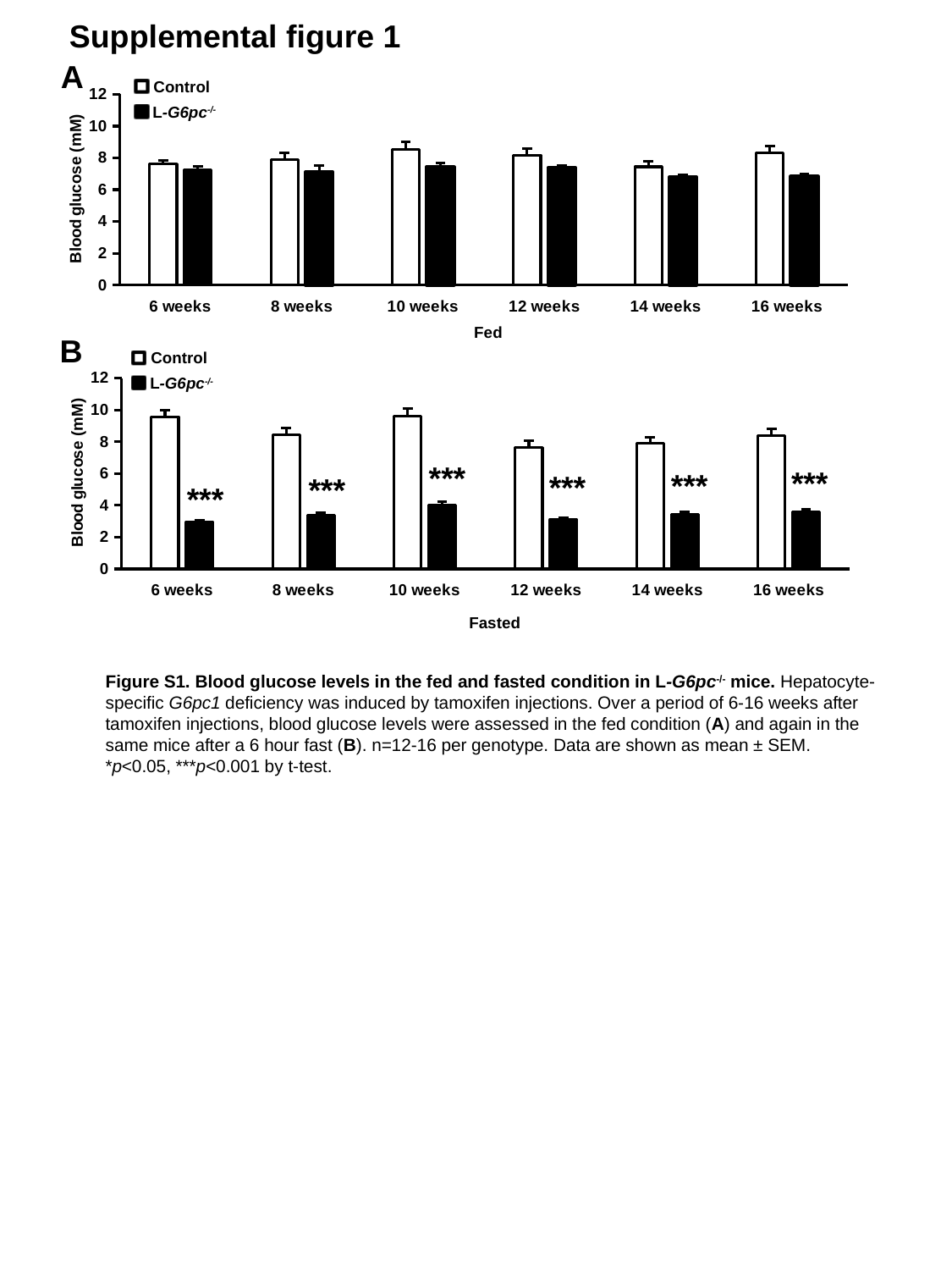

Supplemental figure 1
### Chart
| Category | control ZT1 | L-G6pc-/- ZT1 |
|---|---|---|
| 6 weeks | 7.62 | 7.25 |
| 8 weeks | 7.9 | 7.11 |
| 10 weeks | 8.55 | 7.44 |
| 12 weeks | 8.17 | 7.39 |
| 14 weeks | 7.44 | 6.78 |
| 16 weeks | 8.34 | 6.84 |A
Control
 L-G6pc-/-
### Chart
| Category | control ZT7 | L-G6pc-/- ZT7 |
|---|---|---|
| 6 weeks | 9.56 | 2.93 |
| 8 weeks | 8.43 | 3.4 |
| 10 weeks | 9.63 | 4.0 |
| 12 weeks | 7.64 | 3.09 |
| 14 weeks | 7.93 | 3.44 |
| 16 weeks | 8.38 | 3.61 |Fed
B
Control
 L-G6pc-/-
***
***
***
***
***
***
Fasted
Figure S1. Blood glucose levels in the fed and fasted condition in L-G6pc-/- mice. Hepatocyte-specific G6pc1 deficiency was induced by tamoxifen injections. Over a period of 6-16 weeks after tamoxifen injections, blood glucose levels were assessed in the fed condition (A) and again in the same mice after a 6 hour fast (B). n=12-16 per genotype. Data are shown as mean ± SEM. *p<0.05, ***p<0.001 by t-test.

## Slide 2
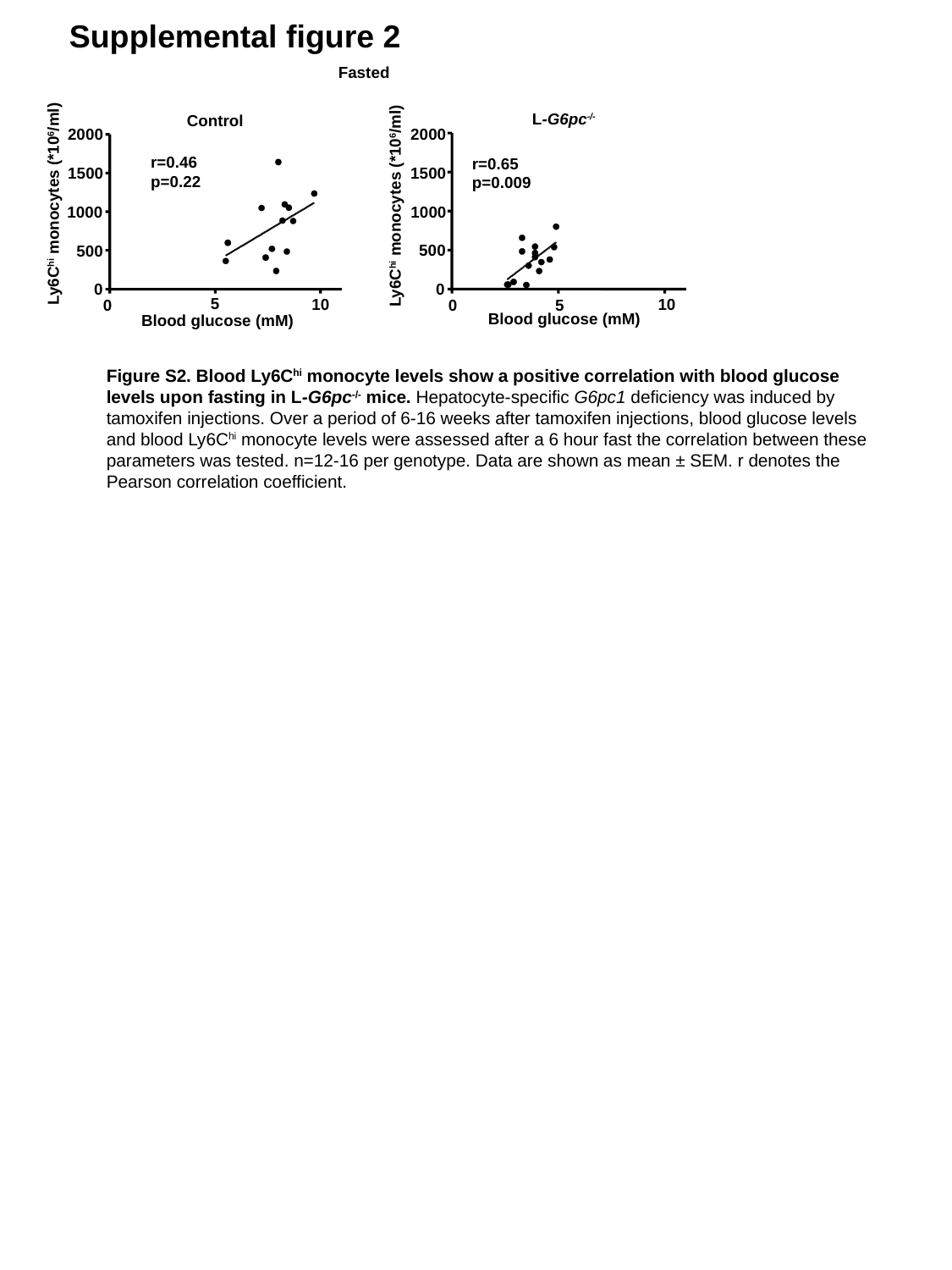

Supplemental figure 2
Fasted
2000
1500
1000
500
0
5
10
0
Blood glucose (mM)
2000
1500
1000
500
0
10
0
5
Blood glucose (mM)
 L-G6pc-/-
Control
r=0.46
p=0.22
r=0.65
p=0.009
Ly6Chi monocytes (*106/ml)
Ly6Chi monocytes (*106/ml)
Figure S2. Blood Ly6Chi monocyte levels show a positive correlation with blood glucose levels upon fasting in L-G6pc-/- mice. Hepatocyte-specific G6pc1 deficiency was induced by tamoxifen injections. Over a period of 6-16 weeks after tamoxifen injections, blood glucose levels and blood Ly6Chi monocyte levels were assessed after a 6 hour fast the correlation between these parameters was tested. n=12-16 per genotype. Data are shown as mean ± SEM. r denotes the Pearson correlation coefficient.

## Slide 3
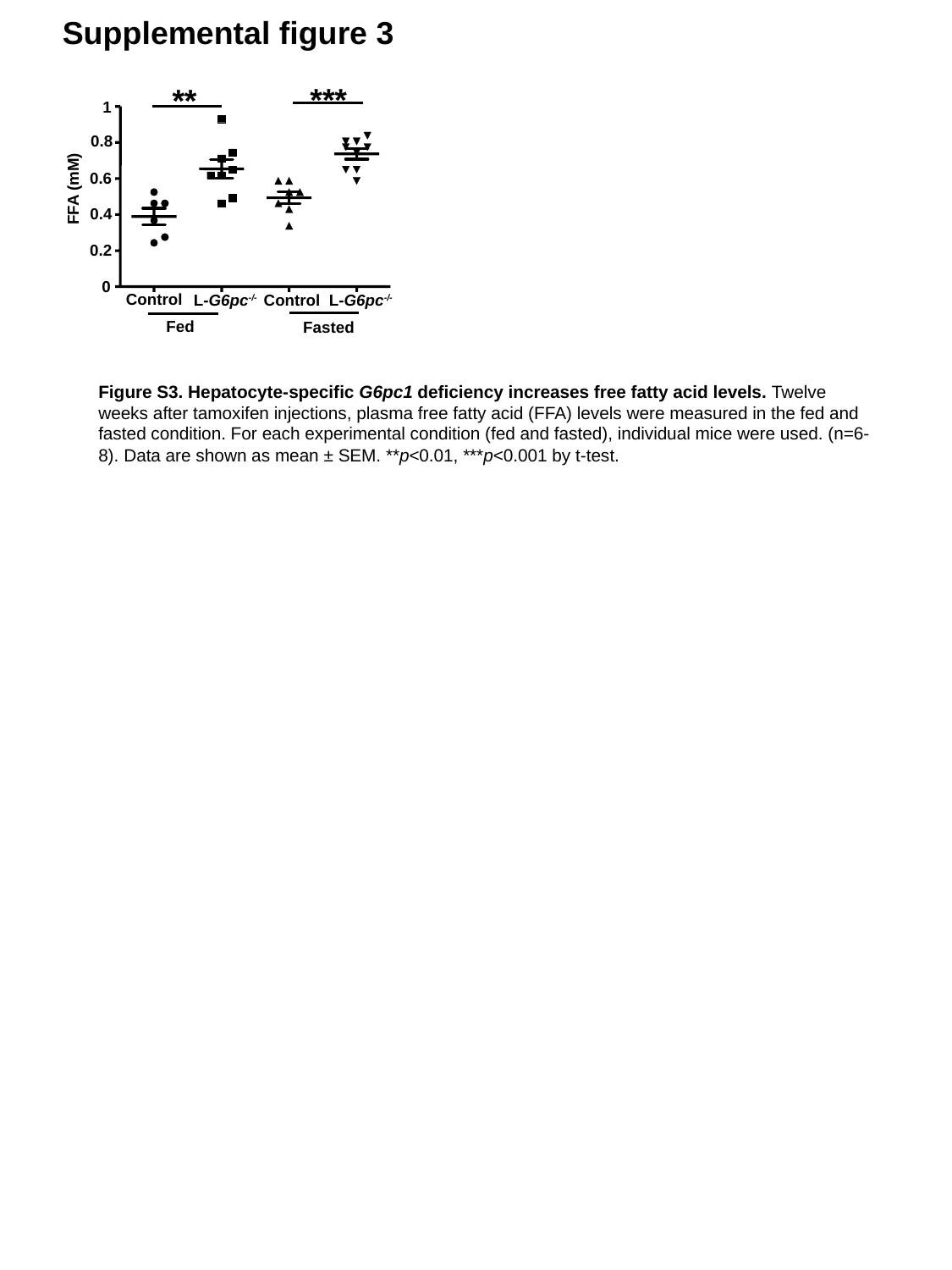

Supplemental figure 3
1
0.8
0.6
FFA (mM)
0.4
0.2
0
***
**
Control
L-G6pc-/-
L-G6pc-/-
Control
Fed
Fasted
Figure S3. Hepatocyte-specific G6pc1 deficiency increases free fatty acid levels. Twelve weeks after tamoxifen injections, plasma free fatty acid (FFA) levels were measured in the fed and fasted condition. For each experimental condition (fed and fasted), individual mice were used. (n=6-8). Data are shown as mean ± SEM. **p<0.01, ***p<0.001 by t-test.

## Slide 4
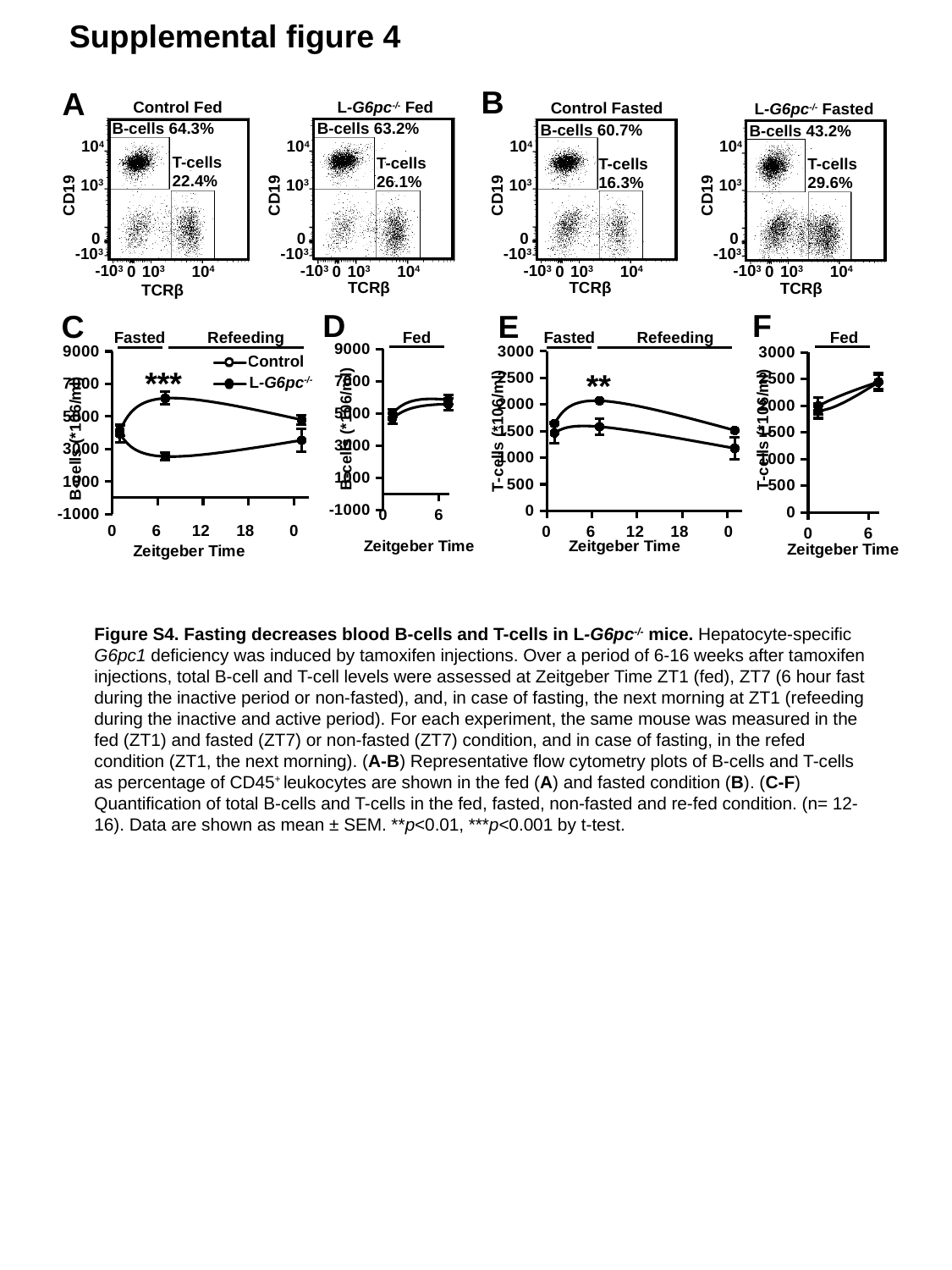

Supplemental figure 4
B
A
Control Fed
L-G6pc-/- Fed
Control Fasted
L-G6pc-/- Fasted
B-cells 63.2%
B-cells 64.3%
B-cells 60.7%
B-cells 43.2%
CD19
104
-103
0
103
-103
104
0
103
CD19
104
-103
0
103
-103
104
0
103
CD19
104
-103
0
103
-103
104
0
103
CD19
104
-103
0
103
-103
104
0
103
T-cells
22.4%
T-cells
26.1%
T-cells
29.6%
T-cells
16.3%
TCRβ
TCRβ
TCRβ
TCRβ
D
F
C
E
### Chart
| Category | control | L-G6pc-/- |
|---|---|---|
### Chart
| Category | control | L-G6pc-/- |
|---|---|---|Refeeding
Fed
Refeeding
Fed
Fasted
Fasted
### Chart
| Category | control | L-G6pc-/- |
|---|---|---|
### Chart
| Category | control | L-G6pc-/- |
|---|---|---|Control
***
**
L-G6pc-/-
0 6 12 18 0
0 6 12 18 0
Figure S4. Fasting decreases blood B-cells and T-cells in L-G6pc-/- mice. Hepatocyte-specific G6pc1 deficiency was induced by tamoxifen injections. Over a period of 6-16 weeks after tamoxifen injections, total B-cell and T-cell levels were assessed at Zeitgeber Time ZT1 (fed), ZT7 (6 hour fast during the inactive period or non-fasted), and, in case of fasting, the next morning at ZT1 (refeeding during the inactive and active period). For each experiment, the same mouse was measured in the fed (ZT1) and fasted (ZT7) or non-fasted (ZT7) condition, and in case of fasting, in the refed condition (ZT1, the next morning). (A-B) Representative flow cytometry plots of B-cells and T-cells as percentage of CD45+ leukocytes are shown in the fed (A) and fasted condition (B). (C-F) Quantification of total B-cells and T-cells in the fed, fasted, non-fasted and re-fed condition. (n= 12-16). Data are shown as mean ± SEM. **p<0.01, ***p<0.001 by t-test.

## Slide 5
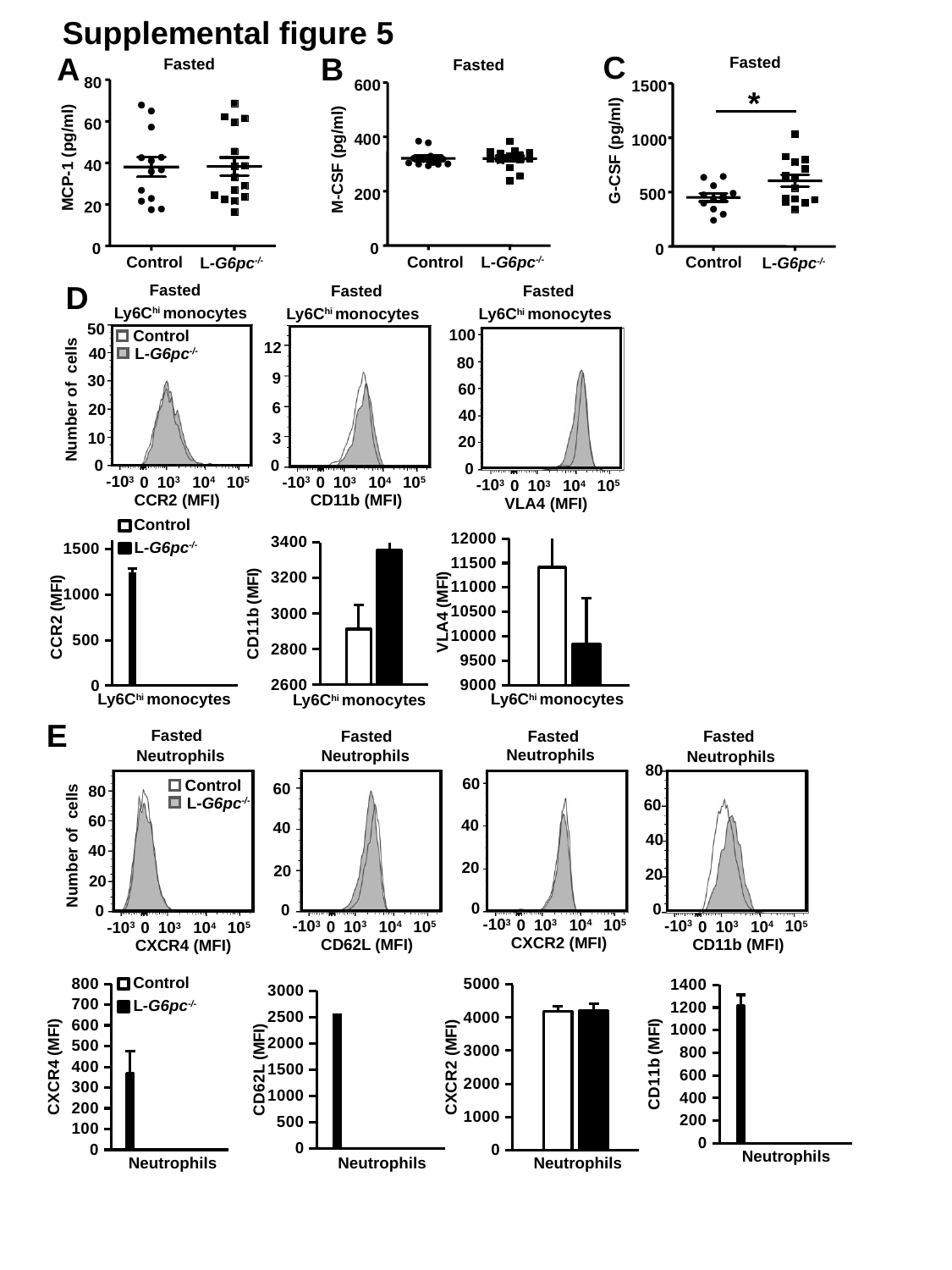

Supplemental figure 5
C
B
A
Fasted
Fasted
Fasted
80
60
MCP-1 (pg/ml)
40
20
0
600
400
M-CSF (pg/ml)
200
0
1500
1000
G-CSF (pg/ml)
500
0
*
L-G6pc-/-
Control
Control
Control
L-G6pc-/-
L-G6pc-/-
D
Fasted
Fasted
Fasted
Ly6Chi monocytes
Ly6Chi monocytes
Ly6Chi monocytes
50
10
0
20
-103
105
104
0
103
Number of cells
CCR2 (MFI)
100
20
0
40
-103
105
104
0
103
VLA4 (MFI)
Control
12
L-G6pc-/-
40
80
9
30
60
0
6
3
-103
105
104
0
103
CD11b (MFI)
Control
### Chart
| Category | Ctrl fast | Cre fast |
|---|---|---|
| Ly6Chi | 11412.57142857143 | 9838.0 |L-G6pc-/-
### Chart
| Category | control | L-G6pc-/- |
|---|---|---|
| Ly6Chi | 1110.4615384615386 | 1242.3333333333333 |
### Chart
| Category | Ctrl fast | Cre fast |
|---|---|---|
| Ly6Chi | 2912.8571428571427 | 3355.875 |Ly6Chi monocytes
Ly6Chi monocytes
Ly6Chi monocytes
E
Fasted
Fasted
Fasted
Fasted
Neutrophils
Neutrophils
Neutrophils
Neutrophils
20
80
0
40
-103
105
104
0
103
Number of cells
CXCR4 (MFI)
80
20
0
40
-103
105
104
0
103
CD11b (MFI)
Neutrophils
60
0
20
-103
105
104
0
103
CXCR2 (MFI)
Control
60
20
0
40
-103
105
104
0
103
CD62L (MFI)
L-G6pc-/-
60
60
40
### Chart
| Category | Ctrl fast | Cre fast |
|---|---|---|
| Neutrophils | 415.7142857142857 | 367.5 |Control
### Chart
| Category | Ctrl fast | Cre fast |
|---|---|---|
| Neutrophils | 4179.428571428572 | 4206.875 |
### Chart
| Category | Ctrl fast | Cre fast |
|---|---|---|
| Neutrophils | 1561.7142857142858 | 1218.375 |
### Chart
| Category | Ctrl fast | Cre fast |
|---|---|---|
| Neutrophils | 2833.8571428571427 | 2509.625 |L-G6pc-/-
Neutrophils
Neutrophils
Neutrophils
Neutrophils

## Slide 6
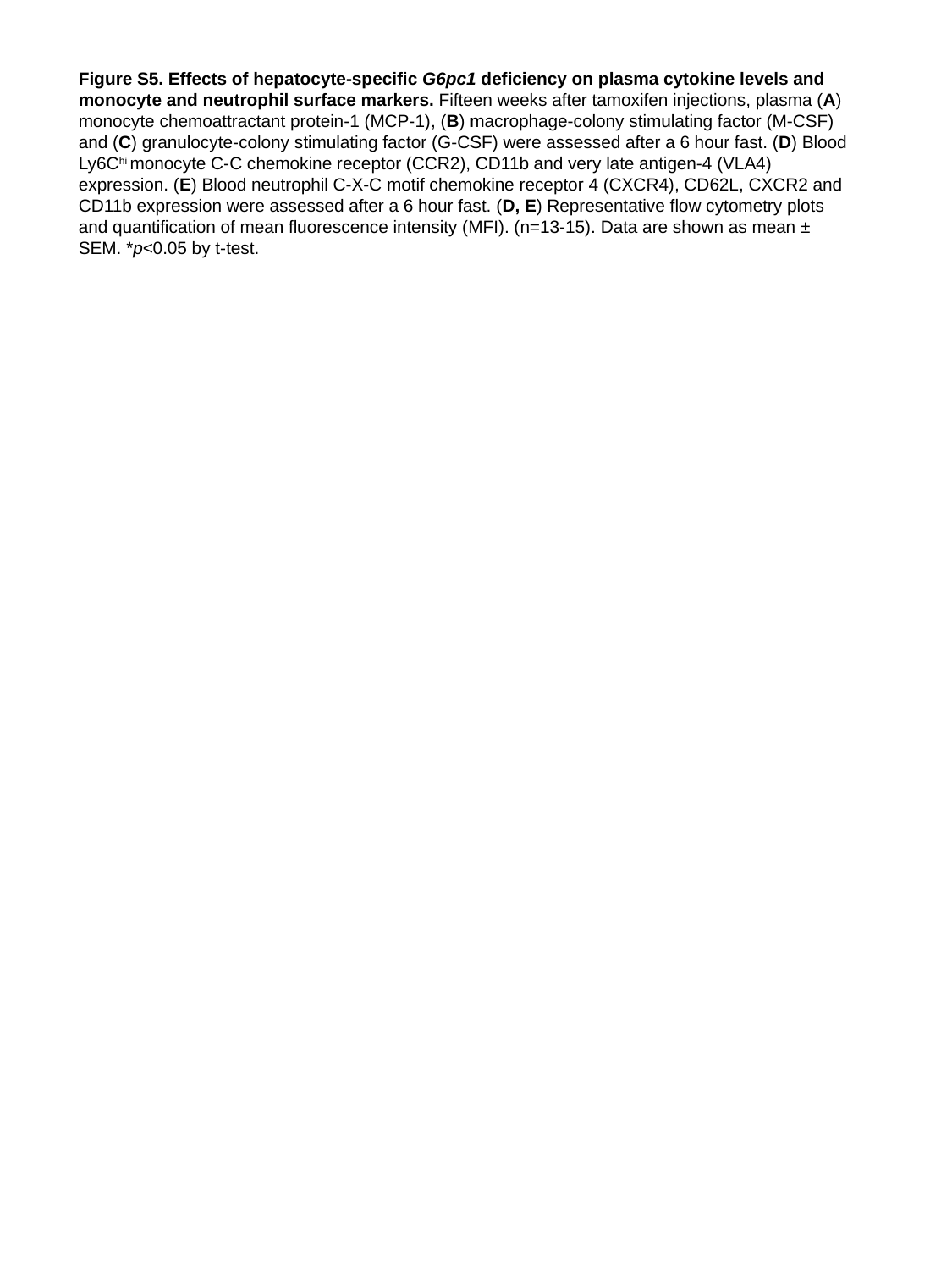

Figure S5. Effects of hepatocyte-specific G6pc1 deficiency on plasma cytokine levels and monocyte and neutrophil surface markers. Fifteen weeks after tamoxifen injections, plasma (A) monocyte chemoattractant protein-1 (MCP-1), (B) macrophage-colony stimulating factor (M-CSF) and (C) granulocyte-colony stimulating factor (G-CSF) were assessed after a 6 hour fast. (D) Blood Ly6Chi monocyte C-C chemokine receptor (CCR2), CD11b and very late antigen-4 (VLA4) expression. (E) Blood neutrophil C-X-C motif chemokine receptor 4 (CXCR4), CD62L, CXCR2 and CD11b expression were assessed after a 6 hour fast. (D, E) Representative flow cytometry plots and quantification of mean fluorescence intensity (MFI). (n=13-15). Data are shown as mean ± SEM. *p<0.05 by t-test.

## Slide 7
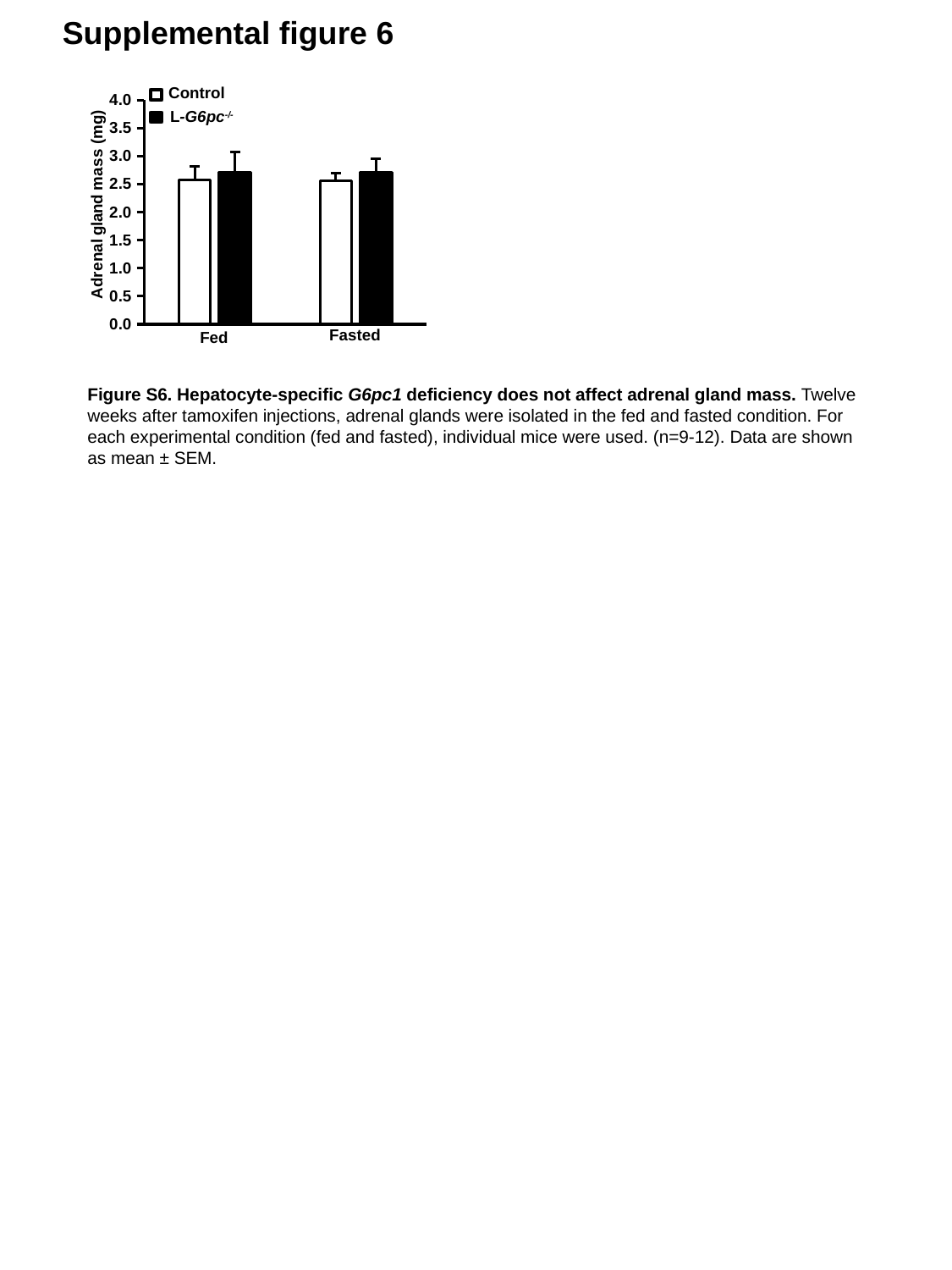

Supplemental figure 6
Control
### Chart
| Category | control | L-G6pc-/- |
|---|---|---|
| ZT1 Fed | 2.5777777777777775 | 2.71875 |
| ZT7 Fasted | 2.5583333333333336 | 2.7125 |
L-G6pc-/-
Fasted
Fed
Figure S6. Hepatocyte-specific G6pc1 deficiency does not affect adrenal gland mass. Twelve weeks after tamoxifen injections, adrenal glands were isolated in the fed and fasted condition. For each experimental condition (fed and fasted), individual mice were used. (n=9-12). Data are shown as mean ± SEM.

## Slide 8
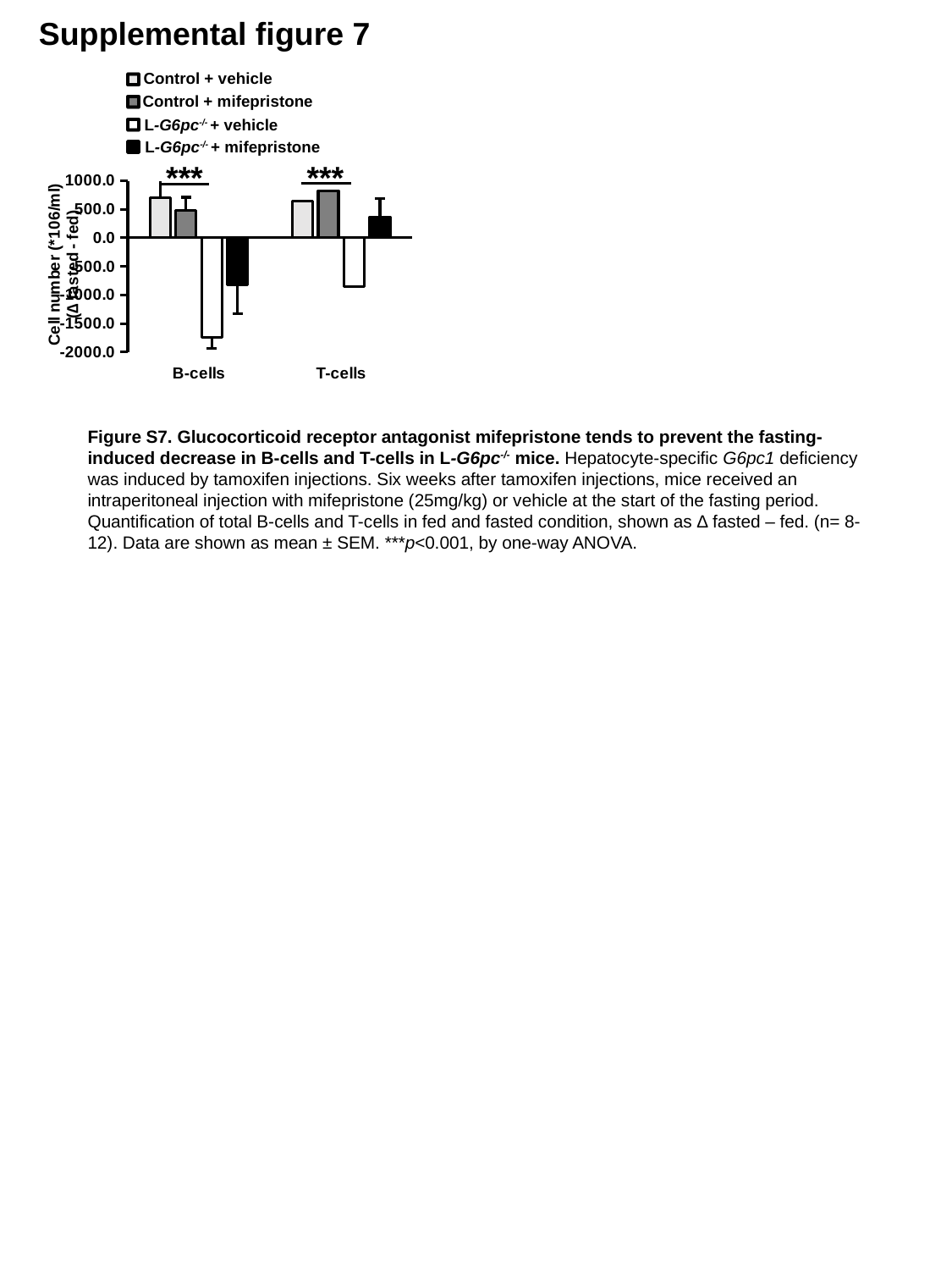

Supplemental figure 7
Control + vehicle
### Chart
| Category | Control + vehicle | Control + mifepristone | L-G6pc-/- + vehicle | L-G6pc-/- + mifepristone |
|---|---|---|---|---|
| B-cells | 700.6398999999997 | 478.02231250000006 | -1738.7945916666665 | -829.7412636363636 |
| T-cells | 638.6722888888889 | 823.6301749999998 | -847.4191666666667 | 363.341190909091 |
Control + mifepristone
L-G6pc-/- + vehicle
L-G6pc-/- + mifepristone
***
***
Figure S7. Glucocorticoid receptor antagonist mifepristone tends to prevent the fasting-induced decrease in B-cells and T-cells in L-G6pc-/- mice. Hepatocyte-specific G6pc1 deficiency was induced by tamoxifen injections. Six weeks after tamoxifen injections, mice received an intraperitoneal injection with mifepristone (25mg/kg) or vehicle at the start of the fasting period. Quantification of total B-cells and T-cells in fed and fasted condition, shown as ∆ fasted – fed. (n= 8-12). Data are shown as mean ± SEM. ***p<0.001, by one-way ANOVA.

## Slide 9
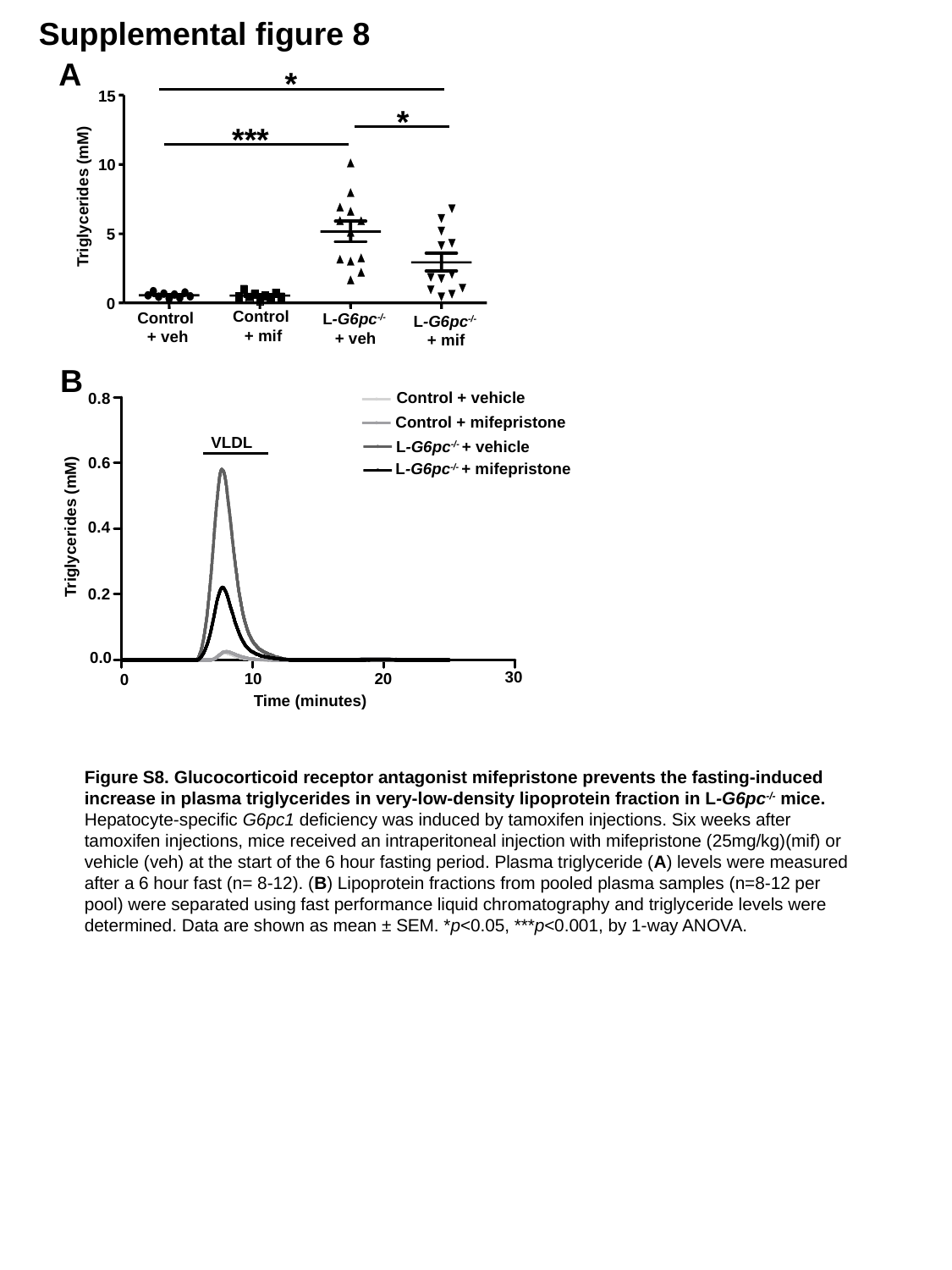

Supplemental figure 8
A
*
15
10
Triglycerides (mM)
5
0
*
***
Control
+ mif
Control
+ veh
L-G6pc-/-
+ veh
L-G6pc-/-
+ mif
B
Control + vehicle
0.8
Control + mifepristone
L-G6pc-/- + vehicle
VLDL
0.6
L-G6pc-/- + mifepristone
0.4
Triglycerides (mM)
0.2
0.0
30
20
10
0
Time (minutes)
Figure S8. Glucocorticoid receptor antagonist mifepristone prevents the fasting-induced increase in plasma triglycerides in very-low-density lipoprotein fraction in L-G6pc-/- mice. Hepatocyte-specific G6pc1 deficiency was induced by tamoxifen injections. Six weeks after tamoxifen injections, mice received an intraperitoneal injection with mifepristone (25mg/kg)(mif) or vehicle (veh) at the start of the 6 hour fasting period. Plasma triglyceride (A) levels were measured after a 6 hour fast (n= 8-12). (B) Lipoprotein fractions from pooled plasma samples (n=8-12 per pool) were separated using fast performance liquid chromatography and triglyceride levels were determined. Data are shown as mean ± SEM. *p<0.05, ***p<0.001, by 1-way ANOVA.

## Slide 10
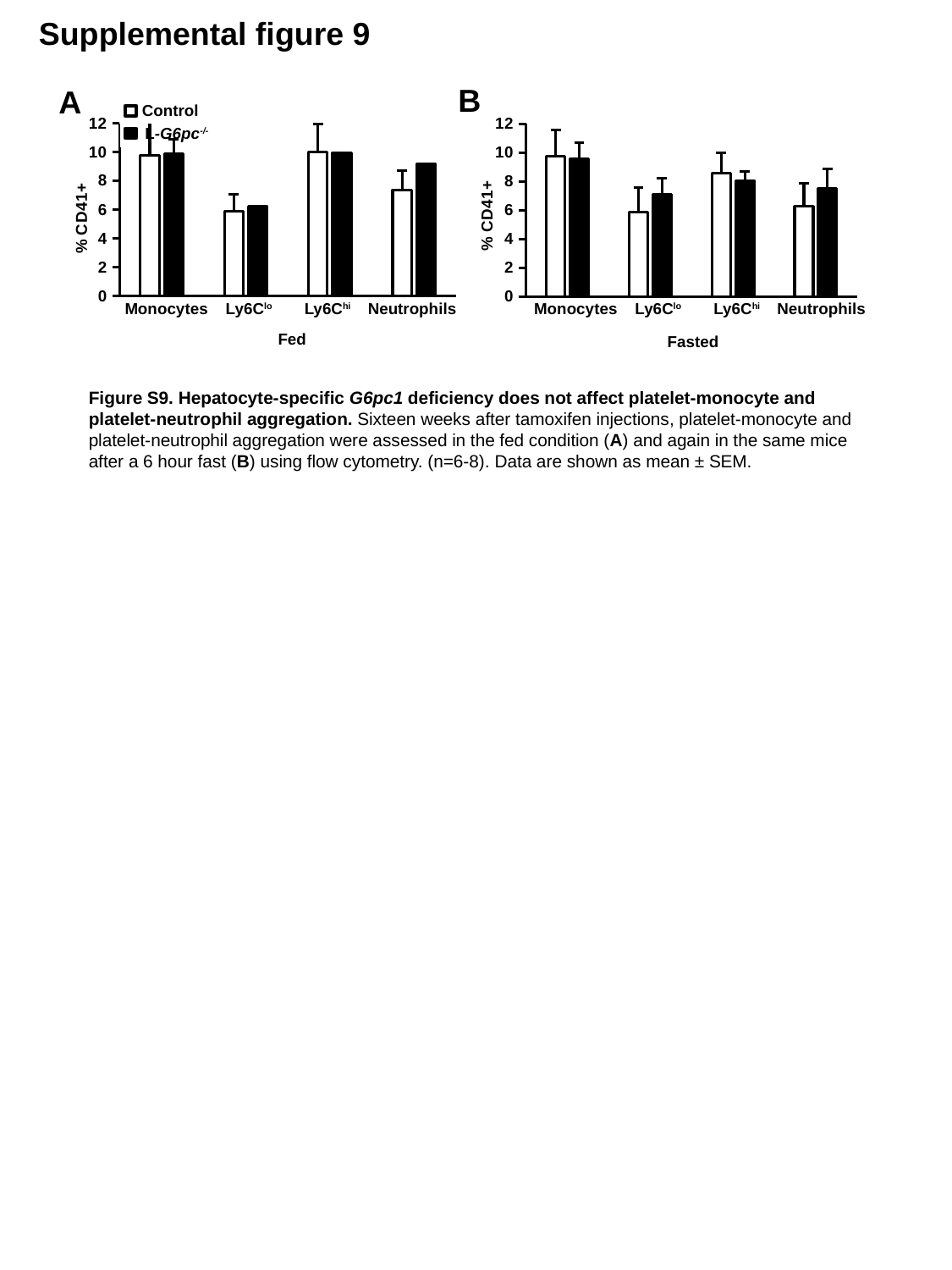

Supplemental figure 9
B
A
Control
### Chart
| Category | control | L-G6pc-/- |
|---|---|---|
| Monocytes | 9.767499999999998 | 9.91625 |
| Ly6Clo | 5.9125 | 6.224999999999999 |
| Ly6Chi | 10.0375 | 9.955 |
| Neutrophils | 7.386250000000001 | 9.162499999999998 |
### Chart
| Category | control | L-G6pc-/- |
|---|---|---|
| Monocytes | 9.758571428571429 | 9.556666666666667 |
| Ly6Clo | 5.884285714285714 | 7.12 |
| Ly6Chi | 8.569999999999999 | 8.066666666666665 |
| Neutrophils | 6.2700000000000005 | 7.5200000000000005 |L-G6pc-/-
Monocytes Ly6Clo Ly6Chi Neutrophils
Monocytes Ly6Clo Ly6Chi Neutrophils
Fed
Fasted
Figure S9. Hepatocyte-specific G6pc1 deficiency does not affect platelet-monocyte and platelet-neutrophil aggregation. Sixteen weeks after tamoxifen injections, platelet-monocyte and platelet-neutrophil aggregation were assessed in the fed condition (A) and again in the same mice after a 6 hour fast (B) using flow cytometry. (n=6-8). Data are shown as mean ± SEM.
